# Low dose TamOxifen and LifestylE changes for bReast cANcer prevention (TOLERANT study): Study protocol of a randomized phase II biomarker trial in women at increased risk for breast cancer

**DOI:** 10.1371/journal.pone.0309511

**Published:** 2024-09-03

**Authors:** Aliana Guerrieri-Gonzaga, Davide Serrano, Patrizia Gnagnarella, Harriet Johansson, Stefania Zovato, Mariateresa Nardi, Matilde Pensabene, Simona Buccolo, Andrea DeCensi, Irene Maria Briata, Luigi Pistelli, Clementina Sansone, Sara Mannucci, Valentina Aristarco, Debora Macis, Matteo Lazzeroni, Gaetano Aurilio, Chiara Arianna Accornero, Sara Gandini, Bernardo Bonanni

**Affiliations:** 1 Division of Cancer Prevention and Genetics, European Institute of Oncology IRCCS, Milan, Italy; 2 Division of Epidemiology and Biostatistics, European Institute of Oncology IRCCS, Milan, Italy; 3 Familial Cancer Unit, Veneto Institute of Oncology IOV IRCSS, Padova, Italy; 4 Division of Breast Oncology, Istituto Nazionale Tumori—IRCCS—Fondazione G. Pascale, Naples, Italy; 5 Division of Medical Oncology, E.O. Galliera Hospital, Genoa, Italy; 6 Wolfson Institute of Preventive Medicine, Queen Mary University of London, London, United Kingdom; 7 Stazione Zoologica Anton Dohrn, Istituto Nazionale di Biologia, Ecologia e Biotecnologie Marine, University of Naples "Federico II", Naples, Italy; 8 Department of Experimental Oncology, IEO, European Institute of Oncology IRCCS, Milan, Italy; PLOS: Public Library of Science, UNITED KINGDOM OF GREAT BRITAIN AND NORTHERN IRELAND

## Abstract

**Background:**

Breast Cancer (BC) prevention strategies range from lifestyle changes such as increasing physical activity and reducing body weight to preventive drugs like tamoxifen, known to reduce BC incidence in high-risk women. Sex Hormone Binding Globulin (SHBG) is related to BC risk due to its ability to bind circulating estradiol at high affinity and to regulate estradiol action. A study protocol is presented based on the assessment of the effect of different interventions such as tamoxifen at 10 mg every other day (LDT), intermittent caloric restriction (ICR) two days per week, lifestyle intervention (LI, step counter use) and their combination on the modulation of SHBG and several other biomarkers associated to BC.

**Methods:**

A randomized phase II biomarker study will be conducted in 4 Italian centers. Unaffected women aged between 18 and 70 years, carriers of a germline pathogenetic variant (*BRCA1*, *BRCA2*, *PALB2*, or other moderate penetrance genes), or with a >5% BC risk at 10 years (according to the Tyrer-Cuzick or the Breast Cancer Surveillance Consortium Risk models) or with a previous diagnosis of intraepithelial neoplasia will be eligible. A total of 200 participants will be randomized to one of the four arms: LDT; LDT + ICR; LI; LI + ICR. Interventions will span six months, with baseline and follow-up clinic visits and interim phone calls.

**Discussion:**

The aim of the study is to verify whether LDT increases circulating SHBG more than LI with or without ICR after 6 months. Secondary objectives include assessing HOMA-index, inflammatory markers, adiponectin/leptin ratio, quality of life (QoL), safety, toxicity, mammographic density, and changes in microbiome composition across groups. The study’s innovation lies in its inclusion of diverse BC risk categories and combination of pharmaceutical and behavioral interventions, potentially enhancing intervention efficacy while balancing tamoxifen’s side effects on QoL, especially menopausal symptoms.

**Trial registration:**

EuCT number:2023-503994-39-00; Clinical trials.gov NCT06033092.

## Introduction

Tamoxifen is an effective agent for breast cancer (BC) prevention in women at increased risk, from familial risk to women with a previous diagnosis of atypical ductal hyperplasia (ADH) and breast intraepithelial neoplasia (IEN) [1].

Therapeutic medicine with tamoxifen or aromatase inhibitors due to their toxicity has a limited clinical application compared to its potentiality [2]. Even during adjuvant therapy, the worsening of quality of life (QoL) can induce the patients to withdraw from the full dose [3]. The first biomarker trials supporting a role for low-dose tamoxifen (LDT) were already published in the 2000’s’ [4, 5]. These studies showed that 5 mg per day of tamoxifen were able to decrease mammographic breast density (MD) in high-risk premenopausal women [4] and to decrease Ki-67 to the same extent as 20 mg per day in a window-of-opportunity trial of 4 weeks of treatment before surgery [5]. Moreover, in a large mono institutional observational study, a dose of 10 mg of tamoxifen every other day significantly decreased the risk of recurrence in women with ductal carcinoma in situ (DCIS) of the breast by more than 30% when compared with no tamoxifen [6]. Based on these findings, our group conducted a phase-III trial (Tam01) in women with excised atypical ductal hyperplasia or breast intraepithelial neoplasia (IEN), showing that 5 mg per day given for 3 years vs placebo decreased the rate of BC events (invasive or IEN) by 52% without increasing adverse events [7]. In particular, there was no excess of endometrial cancer, deep venous thrombosis, or pulmonary emboli in the LDT arm [7]. A 10-year follow up update has confirmed the role of LDT in halving recurrence from non-invasive breast cancer after 7 years from treatment cessation without long-term adverse events [8]. In addition, a recent publication of The Lancet Breast Cancer Commission has recognized LDT as an affordable option of a personalized prevention strategy for risk reduction of hormone receptor-positive breast cancer [9].

Prevention strategies should always start from a healthier lifestyle approach. Evidence has clearly demonstrated the role of obesity and metabolic syndrome as risk factors for major chronic diseases, and lifestyle changes may reduce these risk factors [10]. Furthermore, also the gut microbiota environment may contribute to cancer prevention [11], and its modulation by a healthier diet is a very interesting line of research.

In a cohort of 3,460 postmenopausal women with normal body mass index (BMI) enrolled in the Women’s Health Initiative study, higher body fat levels measured by dual-energy x-ray absorptiometry were associated with increased risk of invasive BC at a median follow-up of 16 years [10].

The majority of BC development and progression is strongly influenced by circulating sex hormones, particularly estradiol. Sex Hormone Binding Globulin (SHBG) is related to BC risk due to its ability to bind circulating estradiol at high affinity [12] and to regulate estradiol action within the cell [13]. SHBG is not just a passive player due to the simple sequestration of circulating estradiol; indeed, the effects on estradiol activity require a specific cell membrane interaction and activation of specific pathways leading to inhibition of estradiol-mediated cell growth and anti-apoptosis [14].

SHBG level can be modulated by diet, physical activity, and drugs. The DIANA study, a randomized dietary intervention trial, showed a significant increase in SHBG over 4.5 months of intervention [15]. Caloric restriction, either continuous or intermittent, leads to an increase in SHBG, which was already evident after one month of intervention [16]. On the contrary, a proinflammatory diet, considered as a BC risk, significantly reduces SHBG plasma levels [17].

Tamoxifen treatment at the standard dose (20 mg/day) induces an increase in SHBG [18]. We have shown that also LDT can increase SHBG with an absolute median change in serum SHBG of 20 nmol/L both in pre and postmenopausal women [19]. Notably, our group showed that SHBG levels were inversely associated with breast neoplastic events [20]. Moreover, breast adipocyte hypertrophy correlates with white adipose tissue inflammation, increased expression of aromatase, the rate-limiting enzyme for estrogen biosynthesis, and higher serum leptin concentrations [21].

Mammographic breast density (MD) is a risk factor for BC. It increases with hormone replacement therapy and may decrease with physical activity and diet [22]. MD reduction after standard tamoxifen dose is an excellent predictor of response in the preventive setting [23]. Remarkably, we have shown that LDT too decreased MD by 20% [4].

Caloric restriction and/or intermittent fasting are emerging approaches to prolong a healthier life by acting on cellular aging and disease risk factors [24, 25].

Fasting typically results in a decrease in serum glucose and depletion of hepatic glycogen, accompanied by a switch to a metabolic mode in which glucose, ketone bodies, and free fatty acid are used as energy sources [24]. Depending on the severity and length of the restriction, fatty acids are metabolized, leading to an increase in circulating ketone bodies and adiponectin and a lowering of circulating leptin, showing a positive effect on metabolic markers [24]. Intermittent energy restriction (25% of the normal caloric intake, 2 days per week) in a group of premenopausal overweight and obese women was effective as traditional energy restriction with regard to weight loss, fasting insulin, insulin resistance, leptin, the leptin/adiponectin ratio, free androgen index, inflammatory markers, lipids, blood pressure, increases in SHBG, Insulin-like Grow Factor Binding Protein (IGFBP)-1 and -2 [16].

Fasting has been found to potentiate the effects of several anticancer treatments, and early clinical studies indicated that patients may benefit from the association between cancer treatment and fasting. Fasting or intermittent fasting diets can reduce serum c-peptide, Insulin-like Grow Factor (IGF)-1, IGFBP-3, and leptin levels while increasing IGFBP-1 [26]. Additionally, it has been shown that, in patients with hormone-responsive BC receiving endocrine therapy and IF, adiponectin, which exerts anti-tumor effects, was increased [27]. Moreover, both fasting and IF prevent tamoxifen-induced endometrial hyperplasia [27]. These results support the rationale to further investigate this association in a randomized biomarker phase II study.

### Objectives

In line with the above-mentioned considerations, the main aim of the study is to verify whether LDT increases circulating SHBG more than LI with or without ICR after 6 months of intervention.

Secondary objectives will test differences by arm for Homeostasis Model Assessment (HOMA)-index, immune and inflammatory markers, lipid profile, Adiponectin/Leptin (A/L) ratio, quality of life (QoL), safety, and toxicity. We will also explore changes in body mass index (BMI) and fat body composition, by Bioelectrical Impedance Vector Analysis (BIVA). We will investigate differences in microbiome composition by arms and the effect of changes in microbiome on QoL considering circulating biomarkers, cytokines, immune modulators, and inflammatory proteins in serum. Finally, we will measure changes in Mammographic Breast Density (MD) by LDT vs LI, with or without ICR: this aim will be performed in a subgroup of participants who will perform baseline mammography (not all the participants will undergo mammography due to younger age).

## Material and methods

### Design and setting of the study

**Fig 1:**
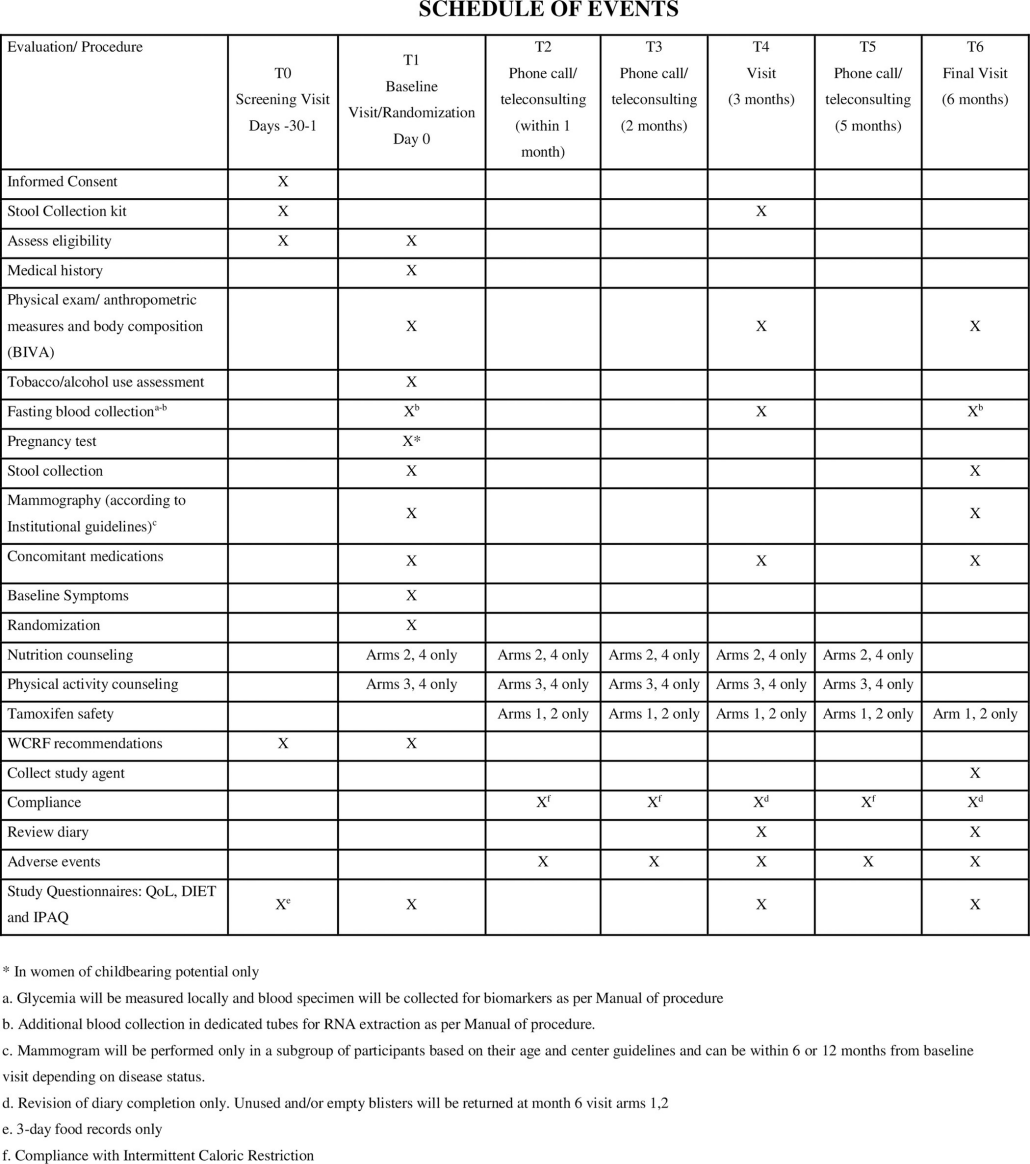
Schedule of events.

The **TOLERANT** (Low dose **T**am**O**xifen and **L**if**E**style changes for b**R**east c**AN**cer preven**T**ion) study is a randomized four-arm phase II intervention trial that will enroll 200 participants at increased risk for BC in four Italian hospitals. Fig 1 details the schedule of events and study procedures at each time point.

Based on the various inclusion criteria and the expertise of each center, women can be enrolled in intensive breast cancer surveillance programs, through mammographic screening programs or through the histopathological review of precancerous breast lesions.

**Fig 2:**
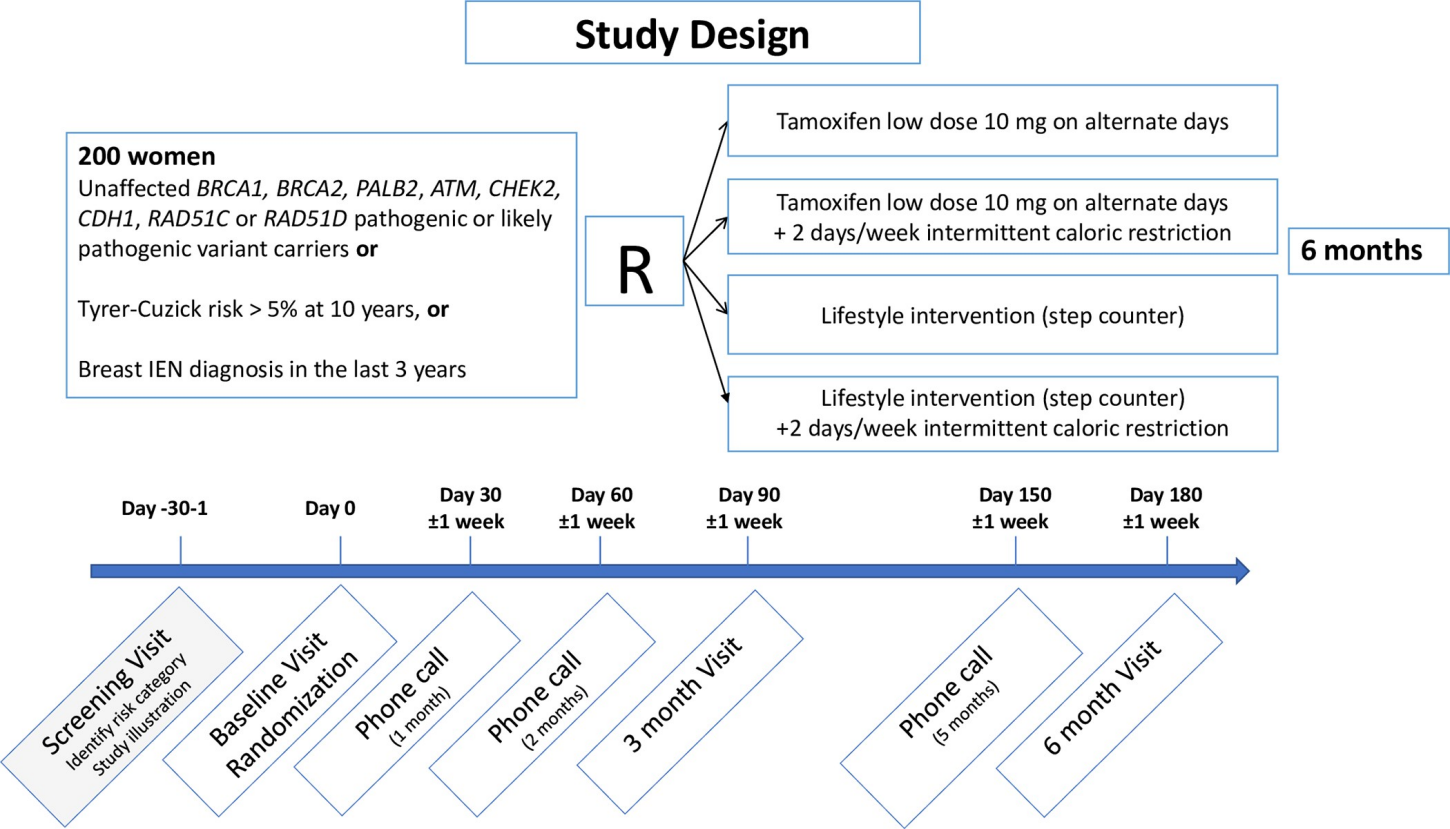
Trial Design population and randomization arms.

Participants informed about the study and will provide written informed consent before enrolling. As shown in Fig 2, participants will be invited to undergo a baseline visit, and after eligibility criteria confirmation, they will be randomly assigned with a 1:1:1:1 ratio to one of the four intervention arms:

*Arm 1*: Low dose Tamoxifen (LDT) i.e., 10 mg every other day;

*Arm 2*: Low dose Tamoxifen (LDT) + Intermittent Caloric Restriction (ICR);

*Arm 3*: Lifestyle intervention (LI) using a step counter;

*Arm 4*: Lifestyle intervention (LI) using a step counter + Intermittent Caloric Restriction (ICR).

Depending on intervention allocation, tamoxifen pills will be dispensed (arms 1 and 2) and dietitians/nutritionists will provide an ICR plan for arms 2 and 4. Participants included in arms 3 and 4, will receive a step counter that encourages them to increase physical activity. All participants will receive diet advice based on international and national recommendations (WCRF.2018; CREA, 2019 https://www.crea.gov.it/web/alimenti-e-nutrizione/-/linee-guida-per-una-sana-alimentazione-2018).

The study is coordinated by the European Institute of Oncology (IEO)—Milan and additional enrolling centers are Galliera Hospital—Genoa, Istituto Oncologico Veneto (IOV)—Padua, and Istituto Nazionale Tumori G. Pascale—Naples.

The study has received approval from the Comitato Etico Territoriale Area Sud-Ovest Veneto (CET-ASOV) and favorable opinion by the Italian Regulatory Authority (AIFA) under the number 2023-503994-39-00 on July 14, 2023. Participants will receive and sign the written informed consent.

The protocol is registered in the Clinical Trials Information System (CTIS) of the European Union (https://euclinicaltrials.eu/search-for-clinical-trials/?lang=en)

The study has been also registered in Clinicaltrials.gov (https://clinicaltrials.gov/study/NCT06033092).

The actual study start date is June 21, 2024 and the expected enrollment completion date is April 2025.

### Sample/participants

#### Main eligibility criteria

Participants must be women between 18 and 70 years old with an ECOG performance status≤1 (Karnofsky >70%) and with the ability to understand and the willingness to sign a written informed consent document.

*Main inclusion criteria are*. Healthy participants carriers of a germline pathogenic/likely pathogenetic variant in at least one of the following genes *BRCA1*, *BRCA2*, *PALB2*, *ATM*, *CHEK2*, *CDH1*, *RAD51C* or *RAD51D*, **or** > 5% BC risk at 10 years, using the Tyrer Cuzick or the Breast Cancer Surveillance Consortium Risk models, **or** with previous diagnosis of intraepithelial neoplasia (surgery for ADH, LCIS, ER positive DCIS) within the last 3 years. A recent negative mammogram or any radiological imaging based on age and center protocol screening baseline visit (six months for healthy participants and 12 months for the IEN group).

*Main exclusion criteria are*. Diagnosis of ER negative (<10%) DCIS, or history of invasive breast cancer; previous treatment with SERMs or any other hormonal treatment for breast neoplasms; BMI < 18.5 Kg/m^2^ and/or Malnutrition Universal Screening Tool (MUST) score ≥2 and/or any current or past eating disorders; any diagnosis of invasive neoplasia, except non-melanoma skin cancer, in the previous 5 years; any tamoxifen contraindications (abnormal liver function, previous ischemic heart disease, endometrial disorder, previous deep venous thrombosis, history of pulmonary embolus, current or suspected glaucoma, retinopathy and cataract); current use of warfarin or other anticoagulant drugs, bilateral mastectomy; pregnancy or desire to become pregnant in the subsequent 9 months after treatment cessation; diabetes or any other clinical condition that at the investigator’s discretion contraindicates the proposed intervention. No hormonal contraception is allowed during study intervention. Non-hormonal methods will be advised for women of childbearing potential (WOCBP).

Women must remain abstinent (refrain from heterosexual intercourse) or use non-hormonal contraceptive methods with a failure rate of <1% per year during the intervention period and, specifically, from 9 months after the final dose of tamoxifen. A woman is considered to be WOCBP if she is post-menarchal, has not reached a postmenopausal state (≥12 continuous months of amenorrhea with no identified cause other than menopause), and is not permanently infertile due to surgery (i.e., removal of ovaries, fallopian tubes, and/or uterus) or other causes determined by the investigator (e.g. Műllerian agenesis). Examples of non-hormonal contraceptive methods with a failure rate of <1% per year include bilateral tubal ligation, male sterilization, and copper intrauterine devices. Hormonal contraceptive (including hormone releasing intrauterine devices) must be interrupted at least 3 months before randomization.

#### Randomization

Once full eligibility has been verified and confirmed, eligibility eCRF has been entered into the dedicated Redcap database web application, the allocated arm will be randomly generated and assigned to the participant.

In order to keep the imbalance in treatments as low as possible, a stratified blocked randomization strategy will be used considering only the relevant prognostic factors. Participants will be stratified according to center and diseases status (healthy high risk versus affected participant).

#### Sample size considerations

Given the results found in our previous studies [19, 20], we considered as main endpoint SHBG (nmol/L) post treatment levels, which has been shown to be associated with risk of first cancer or cancer recurrence. Considering a drop-out rate of 10%, we will have to enroll a total of 200 subjects. Actually, a sample size of overall 180 patients (90 per arm) achieves 80% power to detect a difference of 15 points between the null hypothesis that means of SHBG (nmol/L) post treatment are in both arms 70 and the alternative hypothesis that the mean in the tamoxifen arm is 85, with an estimated group standard deviation of 35 and with a significance level (alpha) of 5% using a two-sided two-sample t-test.

### Procedures and data collection

#### Clinical procedures and evaluations

A schematic description of the study procedures is reported in Fig 1.

Pre-study screening visit/teleconsulting: the health care professional will illustrate the main aim and the trial procedures to the candidate and eligibility will be assessed. Interested participants will be asked to sign the informed consent. A stool collection kit and instruction about baseline sample collection will be provided. The baseline visit will be scheduled within 30 days.

Baseline evaluations include: Physical exam and medical history, vital signs, alcohol and tobacco assessment, anthropometric measurement, body composition by BIVA, QoL assessment, food consumption, and level of physical activity collected through self-administered questionnaires. Pregnancy test will be obtained in women of childbearing potential.

The use of concomitant medications and any baseline symptoms will be recorded. Participants will be instructed about the importance of drug compliance and lifestyle recommendation/instruction based on the randomization arm. All participants will receive a calendar in order to record drug intake, ICR dates, and weekly step counters where appropriate. Participants assigned to the tamoxifen arms will be informed that a pill count will be conducted on their study medication use so that their study medication blisters with remaining tablets must be returned at the last clinic visit. All study interventions will last 6 months.

To maintain high compliance during the 6 months intervention participants will receive three teleconsulting (at T2, T3, and T5) sessions, in order to monitor and reinforce adherence to the proposed intervention, and to record possible adverse effects. At visits T1, T4 and T6 blood samples will be collected together with anthropometric measurements and study questionnaires. The stool will be collected at T1 and T6.

Once the baseline is completed and eligibility criteria have been met, the participants will be randomized to one of the four study intervention arms.

### Description of interventions

#### Low-dose tamoxifen administration

Participants will receive a 7-month drug supply and will be advised to take one tablet (10 mg) every other day (e.g. every odd day at dinner, always at approximately 8 PM).

Details on treatment administration (e.g., dose and timing) should be noted in the source documents and on the eCRF.

#### Intermittent caloric restriction

The so-called “5:2 diet” will be proposed, with 5 days/week at regular energy intake and 2 days/week at 25% of the standard Kcal intake (diet will be restricted at 500–700 Kcal per day, corresponding to a 75% reduction compared to normal intake). Detailed personalized meal plans will be created for each participant, in which portion sizes are reported and possible choices for meal components arranged by food groups to meet the target caloric amount foreseen. Participants will receive a leaflet containing dietary advice and physical activity recommendations to be followed for the study period. During the 2 days of energy restriction, a daily intake of a minimum of 2 L of liquids (water, tea, or infusions) will be strongly recommended to avoid dehydration and side effects of fasting such as hunger and lightheadedness, increased cold sensitivity, weakness, and headaches. Trained dietitians/nutritionists will provide personal nutrition consultations at baseline and during the study period. The intervention will be monitored and reinforced by phone calls at month 1, 2, and 5, and to monitor possible side effects. The first personal consultation within the first month will be used to motivate participants, to monitor compliance, and to consolidate knowledge about dietary programs proposed. Participants will be instructed to complete a fasting diary to monitor the frequency of energy-restricted days. During the 2 days of ICR, women will be advised to avoid tiring and particularly intense activities. If women are used to practice sport, it will be suggested to walk or practice yoga, meditation and light pilates sessions.

#### Lifestyle intervention

Participants will receive personalized advice on healthy lifestyle and the use of a step counter device. These devices tend to encourage both increased physical activity and decreased sedentary time. They are able to record total steps taken, total distance traveled, total time active during the day and the greatest length of time of consistent movements done during the day. A goal of "10.000 steps" per day has been widely promoted and advocated as a strategy for increasing physical activity among able adults. Participants will be asked to wear the step counter during the 6-month intervention period. They could use a smartphone or a tablet application to get feedback on their activity and sedentary time.

Training sessions for nutritionists/dietitians involved in the intervention (lifestyle and caloric restriction) will be organized by IEO as a coordinating center, in order to standardize nutritional counseling and interventions.

#### Toxicity and dose modification

Participants will be asked to maintain the assigned intervention throughout the treatment period. Specifically, for the LDT arm due to the short time of treatment, no dose modification will be applied.

Toxicity will be evaluated using the NCI terminology criteria (CTCAE version 5.0).

Should grade 1 or 2 toxicity occur, the participant will be maintained on treatment irrespective of attribution to the study drug. However, treatment discontinuation should be planned if a persistent/recurrent and intolerable Grade 2 toxicity occurs.

In case of grade 3 toxicity unrelated, unlikely or possibly related to study treatment, participant may remain on treatment as per physician judgment. Women who experience other grade 3 or more severe adverse events will be removed from intervention.

The most commonly reported adverse events (AEs) by early breast cancer patients treated with tamoxifen were (in order of frequency): hot flushes, sweating increase, gynecological disorders, insomnia, and dizziness, musculoskeletal disorders (in particular cramps), peripheral edema, pruritus, reduction in bone mineral density in premenopausal women. At the hematological/biochemistry level, leucopenia, lymphocyte count decrease, and liver enzymes increase.

#### Adherence/compliance

To be compliant, a participant has to take ≥ 75% of the scheduled pills and/or follow 75% of the expected days of intermittent caloric restriction as per the assigned arm. We propose the use of multiple methods of adherence monitoring: subject self-reporting, diary completion, and tablets count.

Diary completion: All participants will receive at each visit a calendar to facilitate tracking and recording of protocol treatment. Each participant will be asked to mark or write Yes/No on the corresponding day of the diary. Based on the treatment assignment, participants are asked to fill the diary and some additional space will be left for the participant’s notes.

A total of 120 pills will be supplied in Arms 1 and 2. Each participant will be asked to return all full and empty blisters.

Should the tablets not be returned, compliance will be calculated from the diary only.

Participants in Arms 2 and 4 will be asked to mark yes/no on the corresponding dates of ICR.

Participants in Arms 3 and 4 will be asked to record the step count number on a weekly basis.

An according-to-protocol subgroup analysis will be restricted to women who are “compliant”, such as participants who have taken ≥ 75% of the pills, for those randomized to tamoxifen and ≥ 75% of the days with the respected caloric restriction, by the reported diary.

#### Study questionnaires

Quality of life will be assessed using the self-administered MenQoL questionnaire (Mapi Research Trust, France) during all visits. This tool is based on 29 items divided into four domains (vasomotor, physical, psychosocial, and sexual). Each item is scored from 1 to 8: 1 means no symptom, 2 indicates the presence of symptoms but not bothersome, and 3 to 8 means an increasing grade of discomfort. The vasomotor domain includes three items to investigate hot flashes and sweating. In the physical domain, there are 16 items covering general symptoms, skin, gastrointestinal, sleeping problems, and urinary symptoms. The psychosocial domain includes seven items, evaluating states of anxiousness, memory, and loneliness. The sexual domain has three items: sexual desire, vaginal dryness, and avoiding intimacy.

Food consumption will be measured at all visits using a short, self-administered questionnaire [28] recently developed to assess adherence to the Mediterranean diet in the Italian population. In this context, it will be used to record the daily or weekly intake of the main food groups over the previous months and the change over the study.

Physical activity will be measured with the IPA Questionnaire (IPAQ) at all visits. This questionnaire consists of questions that record the frequency and duration of mild, moderate, and strenuous exercise performed during free time in the previous 7 days. It measures physical activity and inactivity.

It is a validated self-reported measure of exercise that has been reliably used in previous studies [29]. The total hours per week spent in each activity will be multiplied by the estimated metabolic cost of each activity (metabolic equivalent (MET) value) as determined from the Compendium of Physical Activities [30].

## Specimen collection and handling procedures

Serum, whole blood and stool for evaluation of biomarkers at different timepoints will be collected, processed and stored locally until shipping. Kits for sample collection and identification, specific instructions about specimen handling and storage will be provided by the European Institute of Oncology (IEO) using a specific Manual of Operations and Procedures. Subsequent storage of serum and blood samples will be centralized at the Laboratory of the Division of Cancer Prevention and Genetics, IEO, Milan. The same lab will check sample identification at arrival and coordinate the shipment of samples to the specific laboratories involved in biomarker measurements.

### Blood

Blood samples for serum biomarkers will be collected at baseline, three and six months, and whole blood for RNA samples will be collected at baseline and six months.

Blood samples for serum biomarkers will be drawn under fasting condition (at least 6 hours) preferably between 8 a.m. and 10 a.m. at each visit. A total of 25 ml of blood (5 x 5mL) will be collected into vacuum blood collection tubes containing beads coated with clotting activator for serum separation to be employed for circulating biomarker analysis. Based on specific tubes adopted locally, reference to the manufacture instructions will be followed. Blood will be allowed to clot at room temperature for 30 minutes. Then, it will be spun in centrifuge for 10 minutes. Sarstedt Serum Monovettes tubes require 10 min centrifugation at 2000 x g at room temperature, while BD Vacutainer® SST™ and PST™ gel tubes should be spun at a speed of 1300 x g. Swing-out buckets best option for both systems. After centrifugation, the yellow top layer, which corresponds to serum, is pipetted using the disposable transfer pipettes in 6 even aliquots (about 2 mL each) into the polypropylene cryotubes (Thermo Fisher Scientific), specifically labeled according to the Instructions in the Manual of Operations and Procedures. Tubes are tightly capped and stored in a dedicated– 80°C freezer equipped with a temperature control and temperature log chart or an alarm monitoring system per institutional standards.

Additionally, 2.5 mL whole blood will be drawn into PAXgene® Blood RNA Tubes, for immediate stabilization of intracellular RNA. After blood is drawn into the tube, we will keep the PAXgene Blood RNA Tube upright at room temperature (18−25˚C) for a minimum of 2 hours and a maximum of 72 hours before transferring to freezer (−20˚C). After 24 hours the tubes can be transferred to a −80˚C freezer.

Investigators are advised not to freeze tubes upright in a Styrofoam™ tray as this may cause the tubes to crack.

Specimen collection kits for storage of serum and blood samples will be provided by the Central Laboratory, at the Division of Cancer Prevention and Genetics, European Institute of Oncology in Milan. Briefly, the coordinating center will provide labels and polypropylene cryotubes and racks (Thermo Fisher Scientific) and instructions for specimen handling, processing, labeling, tracking and storage.

Each local study center must be equipped with a– 20°C and -80°C (range -70°C to -80°C) freezer provided with temperature control 24 hours a day, 7 days a week and temperature log charts or an alarm monitoring system, better if electronic. The center should also be equipped with a back-up freezer.

### Stool

Fecal samples will be collected for microbiome analysis at baseline and final visit. Specimens will be collected by patients at home and transported in a collection tube prefilled with preservative liquid, which stabilizes nucleic acids preventing them from being degraded up to 6–8 weeks at room temperature.

Specimen collection kits for storage of fecal samples will be provided by the Istituto Nazionale Tumori Fondazione Pascale di Napoli to each study center.

### Outcomes evaluation

Biomarkers Methods: Serum concentrations of SHBG will be determined by a chemiluminescent immunoassay designed for the IDS-iSYS Multi-Discipline Automated System (Immunodiagnostic Systems Limited, United Kingdom). The lower Limit of Quantitation is 0.30 nmol/L. Serum concentrations of insulin will be determined by a chemiluminescent microparticle immunoassay (CMIA) on the ALINITY *i* System (Abbott Laboratories, Weisbaden, Germany). This assay shows very good agreement with our previously adopted platforms and the assay performance in terms of reproducibility were improved. The sensitivity of the method is ≤ 1.0 μU/mL, and inter-assay coefficient of variation are below 2% at 3 different control levels (low, median, high) produced by Abbott. Inter-assay coefficient of variation of our in-house prepared serum pool (mean: 4.9 μU/mL) is 3.9%. Additionally, we will measure total, LDL, HDL cholesterol and triglycerides levels by the same method. We will calculate the HOMA index, i.e. [fasting insulinemia (mU/L) x glycemia (mmol/L)]/22.5, to be applied as a surrogate index of insulin resistance. Serum adiponectin, leptin and cytokines (IL-6 TNF-alpha) will be measured using an automated platform for immunoassays (ELLA system, ProteinSimple). ELLA is a platform based on a microfluidic technology, that allows to perform automated immunoassays without manual steps in 72 minutes. The inter-assay CVs for adiponectin QCs and our internal control are 4.98% and 6.62%. The inter-assay CVs for leptin QCs and our internal control are 6.77% and 5.35%. The inter-assay CVs of our pooled serum for IL-6 and TNF-alpha were 5.4% (mean 1.26 pg/mL) and 4.6% (mean 6.89 pg/mL), respectively. Serum concentrations of IGF-I and IGFBP-3 will be measured by means of commercially available assays, intended for the quantitative determination in human serum or plasma on the IDS-iSYS Multi-Discipline Automated System. Serum samples from the same participants obtained at different time-points will be run in batches in order to reduce analytical variability. Standards for good laboratory practice will be applied. Monitoring of precision and reproducibility will be performed by processing commercially available control samples and an internal in-house prepared pool of samples in each run. IGFBP1 and IGFBP-2 will be measured by the Human ELISA (Enzyme-Linked Immunosorbent Assay) kit is an in vitro enzyme-linked immunosorbent assay purchased from Abcam Cambridge, UK. We will measure serum concentrations of C-reactive protein by a high-sensitivity turbidimetric method according to the manufacturer’s instructions (Roche Diagnostics, Mannheim, Germany). The test is designed for the automated instrument COBAS INTEGRA 800. The sensitivity for the hsCRP assay is 0.1 mg/L and the intra- and inter-assay coefficients of variation are expected to be 4.1% and 6.4%, respectively for a control sample of 0.423 mg/L.

RNA will be extracted by Qiagen Kit, then subjected to reverse transcription reaction using RT2 first strand kit (Qiagen). Real-Time qPCR performed in triplicate using RT2 Profiler PCR Array (RT^2^ Profiler- PCR Human Innate & Adaptive Immune Responses, Qiagen) for expression of inflammation cell signaling genes. Plates will be run on ViiA7 (Applied Biosystems 384 well blocks), according to standard PCR protocols and online software (http://pcrdataanalysis.sabiosciences.com/pcr/arrayanalysis.php, Qiagen). Cell signaling pathway using CellFate algorithm.

Microbial genomic DNA will be extracted from frozen samples using DNeasy PowerSoil Pro Kit (Qiagen) according to the manufacturer’s instructions, then DNA will be quantified using a Bioanalyzer (Agilent Technologies, CA) and the V3-V4 hypervariable regions of the bacterial 16S rRNA gene will be sequenced on a MiSeq platform (Illumina), 26 enabling taxonomic identification. The 250 bp 16S reads will be processed through QIIME2 (version 2019.7) [31] as follows: (1) Following visualization of demultiplexed samples and the average quality across the reads, quality filtering, dereplicating, and chimera filtering will be performed using the DADA2 [32] plugin within QIIME2, setting the truncation length at 250 bp and the trimming by the length of the V3-V4 primer sequences (—p-trunc-len-f 250,—p-trunc-len-r 250,—p-trim-left-f 17,—p-trim-left-r 2,—p-trunc-q 2), and using consensus as the chimera filtering method; (2) a phylogenetic tree will be generated for downstream core diversity analyses using SATe’-enabled phylogenetic placement (SEPP) [33], which first generates a reference tree—in this case using the SILVA 128 database [34] then inserts 16S sequence fragments into the tree, thus achieving accuracy in phylogenetic tree reconstruction while retaining as much sub-OTU sequences as possible in the tree alpha and beta diversity core metrics will be determined using the qiime diversity command, with the rarefaction depth set to the minimum sequence read output across the samples, after which statistical group comparisons of alpha and beta diversity metrics will be performed, using Kruskal-Wallis for alpha diversity and PERMANOVA for beta diversity; taxonomy classification will be performed using the QIIME feature-classifier classify-sklearn feature, using a Naïve Bayes classifier trained on SILVA 132 99% OTUs full-length 16S rRNA sequences [34], available from the QIIME2 website (https://docs.qiime2.org/2018.11/data-resources/). After filtering the feature count table for unassigned reads and setting a prevalence filter of >50% of the samples, the tables will be collapsed to each taxonomic level (kingdom, phylum, class, order, family, genus, species) and will be exported for further analysis in R. Differential abundance analysis will be performed on the raw counts table using DESeq2 [35], Statistical significance of log2 fold changes will be assessed using the default Wald test with Benjamin-Hochberg p-value correction in DESeq2. Cut-off for all significance tests was set at P < 0.05. Amplicon-based metagenomic approach represents one of the best strategies to obtain a largescale assessment of the taxonomic content of complex samples while containing costs.

Body composition will be assessed using bioelectrical impedance vector analysis (BIVA) (Nutrilab device, AKERN Srl–Italy). BIVA is an accurate method for a quick measurement of body compartments [36, 37]. The direct analysis of the two components of the impedance vector (Z), resistance (R, Ohm) and reactance (Xc, Ohm), allows a semiquantitative evaluation of body composition in terms of body cell mass and hydration status. Data for total body water (TBW), body cell mass (BCM), extracellular water (ECW), fat-free mass (FFM), fat mass (FM) and percentage fat mass (% FM) will be available for all participants and will be used for identifying changes of fat and fat-free mass over the study period.

Moreover, in a subgroup of women, mammographic breast density % change after 6 months will be assessed; the volumetric breast density from digital mammograms will be evaluated by the use of the Volpara software (Volpara Health, UK).

### Statistical consideration

Subject characteristics, information on lifestyle risk factors, diet, and serum biomarkers at baseline will be summarized with descriptive characteristics. Median and interquartile range for continuous variables and absolute and relative frequencies for categorical variables will be presented by arms. Kruskal-Wallis test and Chi-square test (Fisher exact test for sparse data) will be used to assess differences at baseline between arms. Comparisons by affected versus high risk subjects of baseline serum biomarkers will be also carried out using Wilcoxon sum-rank test. We will employ linear regression models to investigate the associations between Tamoxifen treatment, LI and ICR with both post-treatment and changes from baseline of SHBG and all serum biomarkers. We will check whether we need to control for confounding factors and the baseline values. The normality of the models’ residuals will be verified by graphically checking the empirical distribution of the residuals and by looking at Q-Q plots, which compare the cumulative distribution of our data with that of the normal distribution. With multivariate logistic regression, serum biomarkers will be also investigated as categorical variables considering different cut-offs.

To limit the risk of technical bias and to take into account the compositional nature of microbiome data, the normalized taxa abundances will be first transformed with the Centred Log-Ratio (CLR) transformation. The most discriminative taxa associated with treatment arms will be selected by using variable selection approaches adapted for compositional data, like Sparse Least Square–Discriminant Analysis (sPLS-DA) and Coda-LASSO. Multivariate linear regression models will be performed to estimate the association between Tamoxifen, LI, ICR, and post-treatment clr-abundances of taxa, adjusting for confounders. Associations between post-treatment taxa and consumption of specific groups of foods will be also investigated. The level of physical activity will be quantified with the IPAQ questionnaire. Three categories of physical activity (Low, Moderate, High) will be calculated for each patient following the IPAQ scoring protocol [38]. For QoL assessment, we will analyze the four domains of MenQoL questionnaire: vasomotor, psychosocial, physical, and sexual. The overall score of each domain will be calculated by dividing the sum of the domain’s items by the number of items within that domain. Higher domain scores will indicate worse QoL. Associations of the four domains with treatment, LI, ICR and taxa abundances will be estimated considering both post-treatment evaluations (at 3 months and 6 months) and changes from baseline. Multivariate linear or logistic regression will be implemented according to whether the four domains will be analyzed as continuous scales or categorized into classes. The interplay among treatment, serum biomarkers, species (normalized data) associated with treatment arms, diet and QoL will be investigated first with unsupervised methods, like networks based on Spearman’s rank Correlation Coefficient and Canonical Correspondence Analysis (CCA), then with supervised methods for data integration, like block Sparse Least Square-Discriminant Analysis (sPLS-DA) and priority-LASSO hierarchical approaches. To estimate the role of *microbiome* as mediator of treatment arms on SHBG and other biomarkers changes and to investigate its role in mediating the effect of treatments on side effects and QoL, we will perform a mediation analysis based on a counterfactual framework approach. The microbial composition of each patient will be summarized with methods of dimensionality reduction like Principal Component Analysis (PCA) Pathways data will be preprocessed and analyzed as described for the taxonomic data.

The primary analysis will employ an intention-to-treat approach, which includes all participant irrespective of compliance. An according-to-protocol subgroup analysis will be restricted to women who are “compliant”, such as participants who have taken ≥ 75% of the pills, for thus randomized to tamoxifen and ≥ 75% of the days with the respected caloric restriction, by the reported diary.

## Ethical considerations and data protection

Prior to initiating the study and receiving agent, the Participating Organizations must obtain written approval to conduct the study from the appropriate Institutional review Board (IRB). Should changes to the study become necessary, protocol amendments will be submitted to the appropriate IRB for further approval.

All potential study participants will be given a copy of the IRB-approved informed consent to review. The investigator will explain all aspects of the study in lay language and answer all questions regarding the study. If the participant decides to participate in the study, she will be asked to sign and date the informed consent document.

Those who refuse to participate or who withdraw from the study will be treated without prejudice.

Participants will be provided the option to allow the use of biological samples, obtained during testing, operative procedures, or other standard medical practices for further research purposes.

Prior to study initiation, the informed consent document must be reviewed and approved by the appropriate IRB.

Information technology systems used to collect, process, and store study-related data are secured by technical and organizational security measures designed to protect such data against accidental or unlawful loss, alteration, or unauthorized disclosure or access. In the event of data security breach, appropriate mitigation measures will be implemented.

### Data management

The trial will be conducted according to the ICH Good Clinical Practice (GCP) guidelines. Keeping accurate and consistent records is essential to a cooperative study.

The IEO Data Management Office will be responsible of the study database development and data management.

Data for this study will be collected in a REDCap® (Research Electronic Data Capture) database. REDCap was developed specifically around HIPAA-Security guidelines. More information about the consortium and system security can be found at http://www.projectredcap.org/. REDCap is a secure web platform for building and managing online databases and surveys. REDCap’s streamlined process for rapidly creating and designing projects offers a vast array of tools that can be tailored to virtually any data collection strategy. REDCap provides an intuitive user interface that streamlines project development and improves data entry through real-time validation rules (with automated data type and range checks). REDCap also provides easy data manipulation (with audit trails for reporting, monitoring and querying patient records) and an automated export mechanism to common statistical packages (SPSS, SAS, Stata, R/S-Plus). Investigators who have received appropriate institutional research approval (i.e., Institutional Review Board or Institutional Ethics Committee) will be given a web link with a survey where they can enter data about their specific patients. Guidelines about the data collection and to properly enter the data will be developed. The Promoter/Study Coordinator Centre is the legal owner of the collected data and has the right to manage it (including the data collected by the centers involved) in the case of a multi-site study, the sharing of data to any satellite centers is at the discretion of the Coordinator Centre under existing contractual agreements the regulation regarding protected health information (PHI) is also valid in Italy: it is therefore not possible to enter sensitive data of the subjects enrolled in the study or any other data (such as hospital codes/labels/SDO) that can lead to their identity; that’s why when a subject is inserted into the platform, the system assigns her/him a unique identifier. The id/name-last name code decoding is the responsibility of the PI of each experimental center (and should not be shared with the Coordinator Center). Each experimental center will then be the only one to be able to decode the IDs assigned to the subjects managed by its center.

Investigators will access the medical records of their patients, enter required data into the database. The protected health information will not be reused or disclosed to any other person or entity, except as required by law, for authorized oversight of the research project. Future research, that is not defined in this protocol, wishing to access the REDcap database will need institutional review board/ethic review board approval before obtaining access to the REDcap database.

### Clinical data monitoring

Start initiation visits, clinical site monitoring will be performed on in each enrolling site by two clinical monitors who will not be involved in the study conduction to ensure the study is run, recorded, and reported in accordance with the protocol, Good Clinical Practice standards, and any applicable regulatory requirements.

### Protocol deviations

The investigator should document and explain any protocol deviations. The investigator should promptly report any deviations that might have an impact on participant safety and data integrity to the Sponsor and to the IRB/EC in accordance with established IRB/EC policies and procedures. The Sponsor will review all protocol deviations and assess whether any represent a serious breach of Good Clinical Practice guidelines and require reporting to health authorities.

## Considerations/conclusions

Clinical cancer prevention primarily aims to reduce cancer incidence, distinguishing it from screening programs that focus on reducing cancer mortality. By decreasing cancer incidence, we not only alleviate the burden on patients and their families but also significantly cut down the exorbitant costs associated with cancer treatment. Employing multiple approaches for breast cancer prevention enables a more effective and personalized strategy. Addressing the diverse and complex risk factors through genetic counseling, hormonal therapies, lifestyle changes, chemopreventive agents, early detection, and potentially prophylactic surgeries, significantly enhances the overall effectiveness of breast cancer prevention. This multifaceted approach ensures that prevention measures are comprehensive and tailored to each individual’s specific needs and risks.

In the era of precision medicine, it is imperative to consider the complex networks involved in this disease, recognizing how interventions such as lifestyle modifications and drug intake differentially modulate the numerous risk factors. Translational research models, exemplified by biomarker clinical trials, are crucial for better understanding these mechanisms and ultimately validating surrogate biomarkers. This approach is more cost-effective and reduces the pitfalls encountered in the past when conducting large phase III prevention clinical trials predominantly based on epidemiological data.

Despite its short duration, the study could provide significant insights into enhancing the efficacy of interventions while balancing drug side effects on quality of life, particularly regarding menopausal symptoms. By offering a deeper understanding of the interplay between various preventive measures and risk factors, this research holds the potential to refine and optimize breast cancer prevention strategies, ensuring they are both effective and personalized.

## Supporting information

S1 Checklist SPIRIT 2013 checklist: Recommended items to address in a clinical trial protocol and related documents*. (PDF)

S1 File (DOCX)
