# Supplementary material for: Low dose TamOxifen and LifestylE changes for bReast cANcer prevention (TOLERANT study): Study protocol of a randomized phase II biomarker trial in women at increased risk for breast cancer
Source: PLoS One. 2024 Sep 3;19(9):e0309511. doi: 10.1371/journal.pone.0309511 (PMC11371200; doi:10.1371/journal.pone.0309511)
Supplement: S1 File — (DOCX) [file pone.0309511.s002.docx]

**Low dose TamOxifen and LifestylE changes for bReast cANcer prevenTion: a randomized phase II biomarker trial in subjects at increased risk (TOLERANT Study)**

EuCT [2023-503994-39-00](https://euclinicaltrials.eu/ct-sponsor-services/#!/trials/2023-503994-39-00)

UID 3751

**Organization:** **Istituto Europeo di Oncologia**

via Ripamonti 435

20141 Milan, Italy

**Protocol Principal Investigator:** Bernardo Bonanni, MD

**Co-Principal Investigator:** Sara Gandini, PhD

**Organization:** **E.O. Galliera - Ospedale Villa Scassi-Genova ASL3-Regione Liguria**

Mura Cappuccine, 14

16128 Genova Italy

**Local PI:** Andrea De Censi, MD

**Organization:** **Istituto Oncologico Veneto (IOV)**

Via Gattamelata 64

Padova 35128 Italy

**Local PI:** Stefania Zovato, MD

**Organization:** **Istituto Nazionale Tumori G. Pascale**

via Mariano Semmola 53

Napoli 80131 Italy

**Local PI:** Matilde Pensabene, MD

All local Co-Investigators are listed in Appendix A

The study is supported by “Unione Europea Next Generation EU - PNRR M6C2 -Investimento 2.1 Valorizzazione e potenziamento della ricerca biomedica del SSN” (Ref. PNRR-MAD-2022-12376567)”

**SCHEMA**

Low dose **T**am**O**xifen and **L**if**E**style changes for b**R**east c**AN**cer preven**T**ion: a randomized phase II biomarker trial in subjects at increased risk. TOLERANT Study

Selection of eligible participants: either carriers of a germline pathogenetic variant (*BRCA1, BRCA2, PALB2*) **or** with a breast cancer risk >5% at 10 years (according to the Tyrer-Cuzick model) **or** previously treated breast IEN (intraepithelial neoplasia). Women willing to participate will be asked to sign the informed consent and a kit for stool collection will be provided.

**Baseline clinical visit and randomization (T1)**

Physical exam, anthropometric measurements, concomitant medications, fasting blood and stool collection for clinical lab tests and circulating biomarkers, self-reported questionnaires and WCRF recommendations. Gravindex test will be performed in women of childbearing potential.

Based on randomization group, Intermittent Caloric Restriction (ICR) plan and/or Lifestyle Intervention (LI) using step counter and/or drug supply will be provided.

Randomization (stratification by center and high-risk subjects versus previous IEN)

**Intervention (N=200)**

**Arm 1**: tamoxifen 10 mg/ every other day

**Arm 2:** tamoxifen 10 mg/ every other day + ICR

**Arm 3:** LI

**Arm 4** LI + ICR

**Phone call/ teleconsulting (within 1 month**) **(T2)**

**Phone call/ teleconsulting (at 2 months)** **(T3)**

**Three-month clinical visit (T4)**

Physical exam, toxicity assessment, concomitant medications, fasting blood collection for clinical lab tests and circulating biomarkers, self-reported questionnaires, compliance/review pill diary. Kit provision for 6-month stool collection

**Phone call/ teleconsulting (at 5 months) (T5)**

**Final clinical visits (T6)**

Physical exam, toxicity assessment, concomitant medications, fasting blood and stool collection for clinical lab tests and biomarkers, self-reported questionnaires, compliance/review pill diary.

**ENDPOINTS**

**Primary endpoint**:

Post intervention levels of circulating Sex Hormone Binding Globulin (SHBG)

**Secondary endpoints**:

Changes in time of:

Serum biomarkers: SHBG, insulin, glucose, lipid profile, IGF-I, IGFBP-1, -2 and -3, hs-CRP, adiponectin and leptin;

Safety and toxicity;

Quality of life;

BMI and body composition;

Microbiome composition;

Immune modulation by expression of inflammation cell signaling genes.

**Main aim**

Effect of Tam vs LI:

Arm 1 and 2 vs Arm 3 and 4

**Main secondary aim**

Effect of ICR:

Arm 2 and 4 vs Arm 1 and 3

Summary

[1. OBJECTIVES 6](#_Toc138868374)

[1.1 Primary Objective 6](#_Toc138868375)

[1.2 Secondary Objectives 6](#_Toc138868376)

[2. BACKGROUND 6](#_Toc138868377)

[2.1 Clinical Relevance 8](#_Toc138868378)

[2.2 Hypothesis and significance 8](#_Toc138868379)

[2.3 Schedule and duration of intervention 10](#_Toc138868380)

[2.4 Risks and benefits 11](#_Toc138868381)

[3. STUDY DESIGN 11](#_Toc138868382)

[4. PARTICIPANT SELECTION 13](#_Toc138868383)

[4.1 Inclusion Criteria 13](#_Toc138868384)

[4.2 Exclusion Criteria 14](#_Toc138868385)

[5. INTERVENTIONS AND AGENT ADMINISTRATION 15](#_Toc138868386)

[5.1 Interventions and arms description 15](#_Toc138868387)

[5.2 Contraindications 17](#_Toc138868388)

[5.3 Concomitant Medications 17](#_Toc138868389)

[5.3.1 Permitted and prohibited medications 17](#_Toc138868390)

[5.4 Intervention Modification 17](#_Toc138868391)

[5.5 Adherence/Compliance 18](#_Toc138868392)

[6. PHARMACEUTICAL INFORMATION 19](#_Toc138868393)

[6.1 Name of experimental drug 19](#_Toc138868394)

[6.2 Reported Adverse Events and Potential Risks 19](#_Toc138868395)

[6.3 Availability 19](#_Toc138868396)

[6.4 Agent Distribution 20](#_Toc138868397)

[6.5 Agent Accountability 20](#_Toc138868398)

[6.6 Packaging and Labeling 20](#_Toc138868399)

[6.7 Storage 20](#_Toc138868400)

[6.8 Agent Destruction/Disposal 21](#_Toc138868401)

[7. CLINICAL EVALUATIONS AND PROCEDURES 21](#_Toc138868402)

[7.1 Schedule of Events 21](#_Toc138868403)

[7.2 Pre-study screening visit 23](#_Toc138868404)

[7.3 Baseline 23](#_Toc138868405)

[7.4 Evaluation During Study Intervention 24](#_Toc138868406)

[7.5 Three-month visit 25](#_Toc138868407)

[7.7 Off-Agent Criteria 25](#_Toc138868408)

[7.8 Off-Study Criteria 25](#_Toc138868409)

[7.9 Study termination 25](#_Toc138868410)

[7.10 Study and site closure 26](#_Toc138868411)

[8. CRITERIA FOR EVALUATION AND ENDPOINT DEFINITION 26](#_Toc138868412)

[8.1 Primary Endpoint 26](#_Toc138868413)

[8.2 Secondary Endpoints 26](#_Toc138868414)

[9. Collection and Handling Procedures 27](#_Toc138868415)

[9.1. Blood 27](#_Toc138868416)

[9.2 Stool 28](#_Toc138868417)

[9.3 Biomarkers Methods 29](#_Toc138868418)

[9.4. Study specimen and procedure management 31](#_Toc138868419)

[9.5 Shipping Instructions 31](#_Toc138868420)

[9.6 Specimen Banking 32](#_Toc138868421)

[10. REPORTING ADVERSE EVENTS (AE) 32](#_Toc138868422)

[10.1 Adverse Events 33](#_Toc138868423)

[10.2 Serious Adverse Events 35](#_Toc138868424)

[11. STUDY MONITORING 37](#_Toc138868425)

[11.1 Data Management 37](#_Toc138868426)

[11.1.1 Data Protection 38](#_Toc138868427)

[11.1.2 Protocol Deviations 38](#_Toc138868428)

[11.2 Registration/Randomization 38](#_Toc138868429)

[11.3 Case Report Forms 39](#_Toc138868430)

[11.4 Source Documents 39](#_Toc138868431)

[11.5 Record Retention 40](#_Toc138868432)

[12. STATISTICAL CONSIDERATIONS 40](#_Toc138868433)

[12.1 Statistical Plan 40](#_Toc138868434)

[12.2 Randomization/Stratification 41](#_Toc138868435)

[12.3 Statistical analysis 41](#_Toc138868436)

[12.4 Evaluation of Toxicity 42](#_Toc138868437)

[13. ETHICAL AND REGULATORY CONSIDERATIONS 42](#_Toc138868438)

[13.1 Institutional Review Board Approval 42](#_Toc138868439)

[13.2 Informed Consent 42](#_Toc138868440)

[13.3 Other 43](#_Toc138868441)

[14. FINANCING, EXPENSES, AND/OR INSURANCE 43](#_Toc138868442)

[References 44](#_Toc138868443)

[APPENDIX A 44](#_Toc138868444)

[LOCAL CO-INVESTIGATORS 44](#_Toc138868445)

[Appendix B 45](#_Toc138868446)

[Performance Status Criteria 45](#_Toc138868447)

[APPENDIX C 49](#_Toc138868448)

[DIARIO 49](#_Toc138868449)

[Appendix D 54](#_Toc138868450)

[Scheda Farmaco 54](#_Toc138868451)

[Appendix E 55](#_Toc138868452)

[Questionari 55](#_Toc138868453)

[QUESTIONARIO SULL’ATTIVITA’ FISICA 55](#_Toc138868454)

[QUESTIONARIO SULLA DIETA 57](#_Toc138868455)

[QUESTIONARIO SULLA QUALITÀ DI VITA 59](#_Toc138868456)

# OBJECTIVES

## 1.1 Primary Objective

The main aim is to verify whether Low Dose Tamoxifen (LDT) increases circulating Sex Hormone Binding Globulin (SHBG) more than lifestyle intervention (LI) with or without intermittent caloric restriction (ICR) after 6 months.

## 1.2 Secondary Objectives

The secondary aims are:

-to verify whether ICR significantly modulates main and secondary endpoints such as HOMA-index, immune and inflammatory markers, lipid profile, Adiponectin/Leptin (A/L) ratio, quality of life (QoL), Body mass index (BMI), fat body composition, safety and toxicity;

-to verify whether LDT significantly modulates secondary endpoints, such as HOMA-index, immune and inflammatory markers, lipid profile, A/L ratio, QoL, BMI, fat body composition, safety and toxicity;

-to investigate differences in microbiome composition by arms and the effect of changes in microbiome on QoL taking into account circulating biomarkers, cytokines, immune modulators and inflammatory proteins in serum;

- to investigate MD (Mammographic Breast Density) changes by LDT vs LI, with or without ICR. This aim will be performed in a subgroup of participants (not all the participants will undergo mammography due to younger age).

# 2. BACKGROUND

Tamoxifen is an effective agent for breast cancer (BC) prevention in women at-increased risk, including women with a diagnosis of atypical ductal hyperplasia (ADH) and breast intraepithelial neoplasia (IEN)^1^ .

However, its toxicity, from menopausal symptoms to the more serious venous thromboembolic events and endometrial cancer, is a significant problem and may explain the low uptake of tamoxifen as preventive therapy^2^. Moreover, the decreased quality of life (QoL) can determine treatment withdrawal at full dose^3^. To define an optimal dose for the risk/benefit ratio is mandatory to improve preventive therapy. Biomarker data supporting a role for low-dose tamoxifen (LDT) were published in the 2000s’^4, 5^. These studies showed that 5 mg per day of tamoxifen were able to decrease mammographic breast density in high-risk premenopausal women^4^ and to decrease ki-67 to the same extent as 20 mg per day in a window-of-opportunity trial of 4 weeks of treatment before surgery^5^. Moreover, in an observational study, a dose of 10 mg of tamoxifen every other day significantly decreased the risk of recurrence in women with DCIS by more than 30% when compared with no tamoxifen^6^. Based on these findings, our group conducted a phase-III trial (Tam01) in women with excised DCIS or atypical ductal hyperplasia (ADH) or breast intraepithelial neoplasia (IEN), showing that 5 mg/d given for 3 years vs placebo decreased the rate of breast cancer events (invasive or DCIS) by 52% without increasing adverse events^7^. In particular, there was no excess of endometrial cancer, DVT or pulmonary emboli in the LDT^7^.

Out of this study, international guidelines include the use LDT for intraepithelial neoplasia (ASCO^8^, US-PSTF^9^, and NCCN <https://www.nccn.org/professionals/physician_gls/pdf/breast.pdf>).

Breast cancer development and progression are strongly influenced by sex hormones in particular estradiol. SHBG is related to breast cancer risk due to its ability to bind circulating estradiol at high affinity^10^ and to regulate estradiol action within the cell^11^.

SHBG is not just a passive player due to a simple sequestration of circulating estradiol; indeed, the effects on estradiol activity requires a specific cell membranes interaction and activation of specific pathways leading to inhibition of estradiol-mediated cell growth and anti-apoptosis^12^.

SHBG level can be modulated by diet, physical activity and drugs. The DIANA study, a randomized dietary intervention trial, showed a significant increase in SHBG over 4.5 months of intervention^13^. Caloric restriction, either continuous or intermittent, leads to an increase of SHBG, which was already evident after one month of intervention^14^. On the contrary, a proinflammatory diet, considered as a breast cancer risk variable, significantly reduces SHBG plasma levels^15^.

Tamoxifen treatment at standard dose (20 mg/day) induces an increase of SHBG^16^. We have shown that also LDT can increase SHBG with an absolute median change in serum SHBG of 20 nmol/L both in pre- and postmenopausal women ^17^. Notably, SHBG levels were inversely associated with breast neoplastic events^18^.

Mammographic breast density (MD) is a risk factor for BC. It increases with hormone replacement therapy and may decrease with physical activity and diet^19^. MD reduction after standard tamoxifen dose is an excellent predictor of response in the preventive setting^20^. Notably, we have shown that LDT decreased MD by 20%^4^.

Evidence has clearly demonstrated the importance of BMI and metabolic syndrome as risk factors for major chronic diseases. Lifestyle changes may reduce this risk factor^21^, and microbiota modulation by a healthier diet may contribute to cancer prevention^22^.

Excess weight or weight gain during adult life increase the risk of several diseases, including BC^23^. In a cohort of 3460 postmenopausal women with normal BMI enrolled in the Women's Health Initiative randomized clinical trial, higher body fat levels measured by dual-energy x-ray absorptiometry were associated with increased risk of invasive BC at a median follow-up of 16 years ^21^.

Breast adipocyte hypertrophy correlates with white adipose tissue inflammation, increased expression of aromatase, the rate-limiting enzyme for estrogen biosynthesis, and higher serum leptin concentrations^24^.

Caloric restriction and/or intermittent fasting (IF) are emerging approaches to affect health span by acting on cellular aging and disease risk factors^23, 25^

Fasting typically results in a decrease in serum glucose and depletion of hepatic glycogen, accompanied by a switch to a metabolic mode in which glucose, ketone bodies and free fatty acid are used as energy sources^25^. Depending on the severity and length of the restriction, fatty acids are metabolized, leading to an increase in circulating ketone bodies and adiponectin and a lowering of circulating leptin, showing a positive effect on metabolic markers^25^. Intermittent energy restriction (25% of the normal caloric intake, 2 days per week) in a group of premenopausal overweight and obese women was effective as traditional energy restriction with regard to weight loss, fasting insulin, insulin resistance, leptin, the leptin/adiponectin ratio, free androgen index, inflammatory markers, lipids, blood pressure, increases in SHBG, IGFBP1 and -2^26^.

Fasting has been found to potentiate the effects of several anticancer treatments, and early clinical studies indicated that patients may benefit from the association of cancer treatment and fasting. Fasting or IF diets can reduce serum c-peptide, IGF1, IGFBP3 and leptin levels, while increase IGFBP1^27^. Additionally, it has been shown that, in patients with hormone responsive BC receiving endocrine therapy and IF, adiponectin, which exerts anti-tumor effects, was increased. Moreover, both fasting and IF prevent tamoxifen-induced endometrial hyperplasia ^28^. These results support the rationale to further investigate this association.

2.1 Clinical Relevance

Our group has intensively investigated the use of LDT as preventive medications to improve the benefit/risk ratio to offer safe and effective drugs to women with an increased risk for breast cancer. Recent data show that low dose tamoxifen is the most popular choice of preventive therapy in women with preneoplastic lesions, with low discontinuation rates at 1 year compared other preventive drugs^29^. Our aim is to improve also primary prevention for women who never had a premalignant diagnosis, the innovation in this program is the variety of risk categories included, and the combination of a pharmaceutical and a behavioral intervention. This association may improve the efficacy of the intervention and ameliorate the QoL balancing tamoxifen potential side effects, specifically the menopausal symptoms.

## 2.2 Hypothesis and significance

Despite the strong evidence of tamoxifen efficacy for BC prevention, its use in clinical practice is still low. Fear of side effects hampers its uptake by high-risk women who could benefit from tamoxifen preventive activity^2^.

Among the strategies to overcome tamoxifen side effects, we have intensively investigated alternative low doses of tamoxifen in phase II and phase III studies. In the phase III studies, we have been able to document that serious adverse events were not significantly different from placebo. However, menopausal symptoms were still greater in the tamoxifen group^7, 30^. In our previous phase II and III studies we used 5 mg per day, but this dosage is not commercially available. In our monoinstitutional observational study by Guerrieri-Gonzaga et al^6^, in order to maintain the dose of 5 mg per day, we prescribed tamoxifen 10 mg every other day since commercial tamoxifen pills can not to be split in half. To adopt a practical approach, we chose this schedule to use the commercially available formulation.

A second point to be improved to reduce the resistances by high-risk women as well as physicians to discuss tamoxifen therapeutic prevention is to identify patients who may have the largest benefit from tamoxifen intervention and the ones who may be more susceptible to menopausal symptoms.

We have a large experience in clinical biomarker trials and we also investigated biomarkers to identify high cancer risk subjects. In a metanalysis in postmenopausal women, high SHBG level was significantly associated with decreased risk of breast cancer (p<0.001)^31^. We showed in a pre-perimenopausal population that the median value of SHBG was significantly lower in women operated for IEN/T1mic compared to healthy women (with a Gail-risk 5 years ≥ 1.3 %). Furthermore, these populations were followed up to 10 years and the incidence of breast cancer events was inversely associated with the SHBG levels (P = 0.018)^18^.

- *Given the above considerations:* we will assess SHBG modulation, comparing LDT with LI, with or without ICR. Several other BC risk biomarkers will be evaluated.

LI and diet have been extensively studied as modifiable risk factors for BC development. Nearly 25% of BC can be attributed to lifestyles (overweight/obese women and/or sedentary attitude). Our group carried out a trial in BC survivors to verify the role of LI intervention on BMI change and QoL^32^. We enrolled 260 women, randomized into one of the four intervention arms: Dietary Intervention (DI); Physical Activity Intervention (PAI); Physical Activity and Dietary Intervention (PADI); Less Intensive Intervention (LII). Higher percentages of women included in the DI and PADI arm reached the objective of the trial (weight reduction >5%), 37.5% and 36.7% respectively. Significant decrease of body weight, waist and hip circumferences were observed at 6-month in the four arms. The mean relative change in body weight was more marked in the DI and PADI arms (-4.7%±5.0% and -3.9%±4.5%, respectively) and persisted over time (at 12 and 24 months) where counseling was mainly focused on the dietary intervention (dietetic component). The intervention had also an effect on the blood glucose level with a significant reduction in the whole sample and in the PADI arm (-2.5± 7.8, p-value 0.03) (Gnagnarella et al, submitted).

Our hypothesis is that LI and adherence to a healthy diet and implementation of physical activity play a key role in BC prevention. Several studies have shown that diet and caloric restrictions bring to favorable changes in adiponectin, leptin, CRP, IL-6, insulin and SHBG, all of which are risk biomarkers involved in breast carcinogenesis ^33^.

Side effects and QoL are crucial issues for the uptake of cancer prevention programs. Increase in menopausal symptoms can be a withdrawal reason even in BC adjuvant therapy and usually these patients are even less likely to be engaged in health-promoting activities. Overall, it has been shown that undertaking moderate to vigorous physical activity is associated with fewer menopausal symptoms, especially for cognitive and sleeping disturbances^34^. In parallel, low-fat diet intervention showed a reduction in vasomotor symptoms^35^

Preclinical and some clinical studies indicate that fasting has potential benefits on cancer diseases free and overall survival while reducing therapies side effects^36^. Similarly, cancer risk biomarkers (reducing IGF-1, glucose, CRP and increasing IGFBP-1) might be favorably modulated by caloric restriction^37^. Overall ICR “5:2 diet” can be equivalent to continuous calorie restriction for weight reduction and prevention of metabolic diseases^38^. Since there is an inverse association between BMI and SHBG^39^, ICR could improve this balance. Furthermore, it has been shown that caloric restriction reduce leptin, and, at lesser extent, increased adiponectin, and the effect was significant for both adipokines when the restriction was ≤ 50% of the normal caloric intake^40^.

- *Given the above considerations*: we will assess whether a more watchful change in lifestyle making use of a step counter device (lifestyle intervention, LI) and/or ICR will improve the risk biomarkers modulation, in particular adiponectin/leptin ratio. Furthermore, the modulation of QoL will also be evaluated.

Microbiome may affect tumor initiation and progression through direct effects by toxic metabolites and indirectly through manipulation of the immune system. It can also determine response to cancer therapies and predict disease progression and survival. In particular, we showed that for colorectal cancer the microbiome is a reproducible biomarker and may have a role in cancer prevention^41^. We have recently shown that microbiome taxa are associated with diet and lifestyle, markers of inflammation, dysbiosis, adiponectin and 25OHD levels and they could be a mediator of diet for cancer risk. Molecular tests are also becoming more affordable and widely used, and fecal microbiome signature could provide an alternative or a second level test for personalized screening and prevention medicine programs. Recently, gut microbiota dysbiosis has been shown to play a role also on BC incidence and prognosis^42^. The microbiota may act through several pathways including host immunity, chronic inflammation, oncogenic signaling, hormonal pathways.

- *Given the above considerations*: we will assess the microbiota profile before and after intervention and we will correlate microbiota with several biomarkers of immune system function, inflammation, hormones and lipid profile.

Finally, carcinogenesis and immune system are highly correlated. Immune cell types may act as both cancer-suppression and cancer-progression^43^. An increased percentage of immunoregulatory T (Treg) cells in the blood and tumors has been showed in breast cancer patients^44^. In addition, in advanced BC stages an increase of immunosuppressive cells was observed regardless of ER status^45^. Another study showed that gene expression profile detectable in the peripheral leucocytes (low CD163, CXCR4, and high THBS1 expression) identify triple negative breast cancer with a worst prognosis^46^.

- *Given the above considerations*: we will assess gene expression profile pre and post intervention from Peripheral Blood Mononuclear Cells (PBMC). The gene expression will be correlated will the other biomarkers in particular the microbiome that can directly affect the immune system.

## 2.3 Schedule and duration of intervention

Duration of intervention: Phase II studies have the advantage to be able to reach their endpoint in a short period of time. Drugs biological effect can be seen within days if not hours. SHBG modulation by tamoxifen can be seen already at two weeks as reported by Birzniece et al. ^47^. Their paper showed a significant SHBG increase for both 10 and 20 mg already after two weeks of treatment. Our presurgical study reported specifically for 5 mg per day of tamoxifen a modulation of SHBG from nmol/L 65.3 (52.5 to 78.1) to 70.9 (60.2 to 81.7) in four weeks (significant trend compared with the control arm) ^5^.

We have published a paper with tamoxifen a 10 mg a week where the increment of SHBG was seen at three months and was stable at six and 12 months (published data on the ratio Estradiol/SHBG, row for SHBG data alone not published) ^48^. Finally, a subsequent prevention study with low dose tamoxifen measured SHBG only at 12 months ^17^. With all these observations we decided to set at six month our intervention, sufficient to see the modulation of the primary endpoint but also useful to see effects of the LI and ICR arms and quality of life.

Drug schedule: Our main randomized trial used a non-commercially available formulation of 5 mg per day. Tamoxifen has a long half-life^49^, and we explored also lower dose likewise 1 mg per day and 10 mg per week^48, 50^. All these lower doses/schedules have shown that tamoxifen maintains biological effects, including SHBG modulation^48^. Furthermore, this specific schedule has been already used for intraepithelial patients with a clinical benefit decrease by 30% of new breast events (either in situ or invasive)^6^. To have a pragmatic approach and to reduce expenses we choose the commercially available dose of 10 mg every other day (equivalent to 5 mg per day) to avoid a study-specific drug formulation.

## 2.4 Risks and benefits

Anticipated potential side effects are clearly different based on arm allocation. Tamoxifen toxicity is reported in the pharmaceutical information, section 6.2, and the full list is reported in the SmPC document. In regard to the lifestyle intervention, potential side effects are illustrated in section 10.1. Briefly, due to the short period of the study intervention we do not expect many adverse events. The inclusion and exclusion criteria will allow to select participants with no potential concomitant risk factors. Up to 80% of women may experience menopausal symptoms with tamoxifen at 20 mg per day. We have shown that side effects with LDT are only marginally increased compared to placebo^7^. For the LI intervention, in particular during the two days/week of ICR arms, fatigue, dizziness, tiredness, loss of energy, feeling of cold hands, and lack of concentration, can occur, but the personalized counseling provided over the course of the study should result in a reduction of these side effects. Given that the study is a phase II clinical trial, we cannot expect an immediate clinical benefit, although an improvement of quality of life and overall health status even on a short-time period can be achieved and, eventually, participants may maintain a healthier behavior for a longer period of time.

# 3. STUDY DESIGN

The TOLERANT (Low dose **T**am**O**xifen and **L**if**E**style changes for b**R**east c**AN**cer preven**T**ion) study is a randomized four-arm phase II intervention trial that will enroll 200 participants at high risk of breast cancer in four Italian centers (Milan, Genoa, Padua and Naples).

Potentially eligible women will be informed about the study and will provide signed informed consent before participation. At baseline, participants will be screened to confirm eligibility and will be randomly assigned (1:1:1:1) to one of the four intervention arms (Figure 1):

*Arm 1:* Low dose Tamoxifen (LDT) i.e 10 mg every other day;

*Arm 2:* Low dose Tamoxifen (LDT) + Intermittent Caloric Restriction (ICR);

*Arm 3:* Lifestyle intervention (LI) using a step counter;

*Arm 4:* Lifestyle intervention (LI) using a step counter + Intermittent Caloric Restriction (ICR).

Figure 1


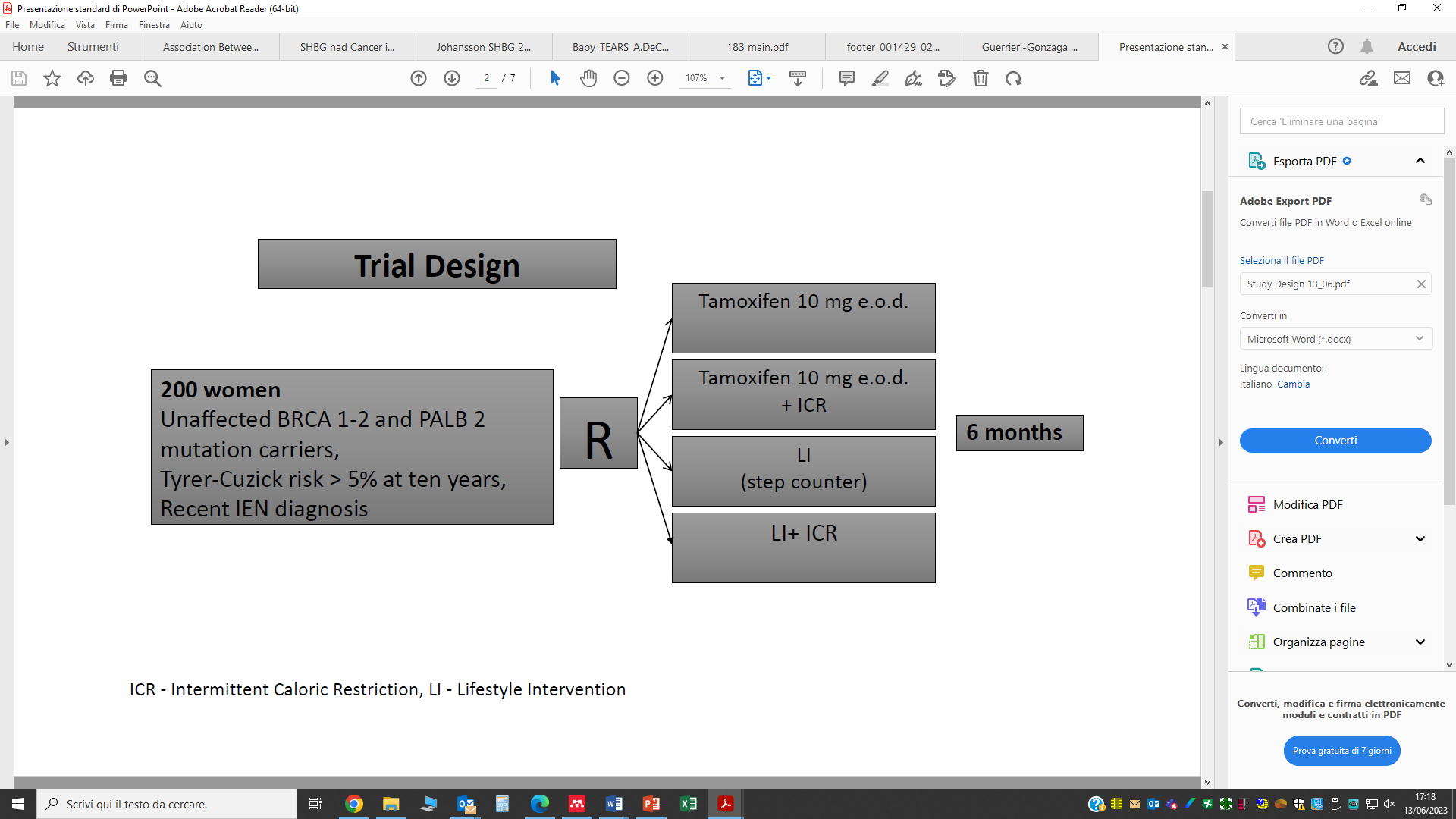


Stratification: center and disease status (high risk vs previous IEN)

A schematic description of the study procedures is reported in figure 2.

Figure 2


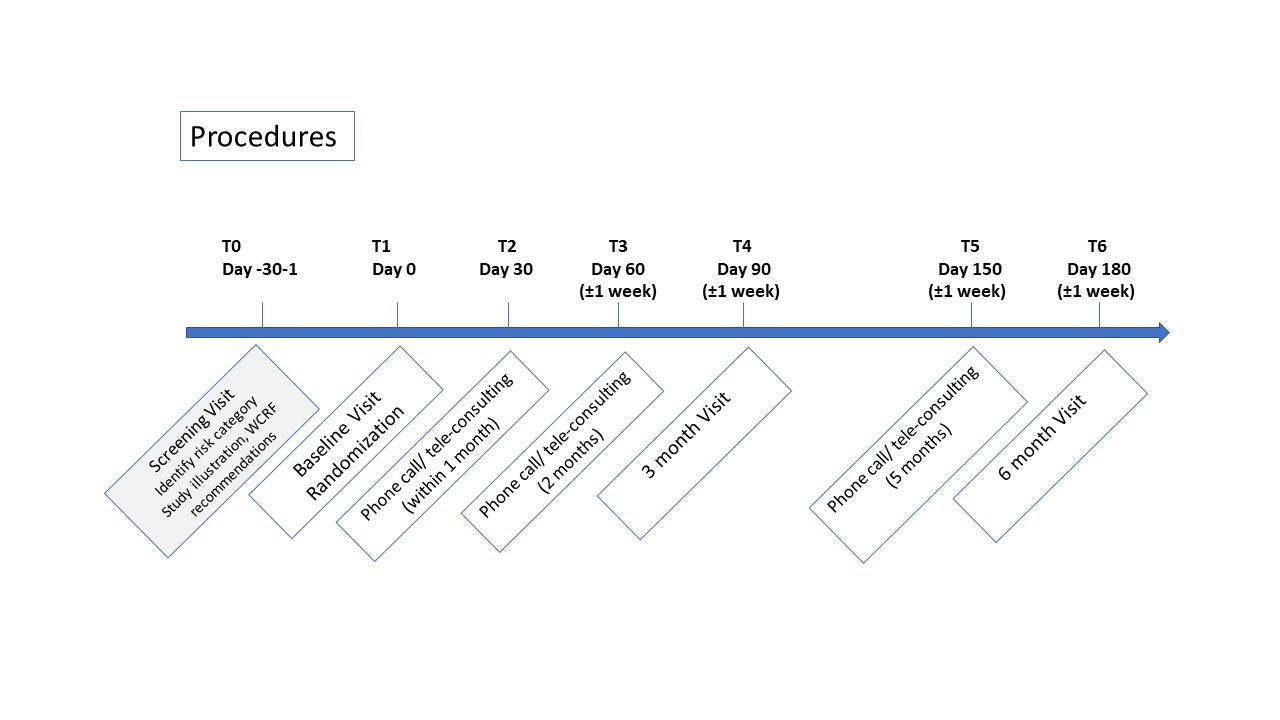


Trained staff including dietitians/nutritionists will provide advices based on international and national recommendations (WCRF.2018; CREA, 2019 https://www.crea.gov.it/web/alimenti-e-nutrizione/-/linee-guida-per-una-sana-alimentazione-2018) at baseline to all participants. Depending on intervention allocation, dietitians/nutritionists will provide a caloric restriction plan to Arm 2 and 4. Participants included in Arm 3 and 4, will receive a step counter that encourages both increased physical activity and decreased sedentary time. Depending on intervention allocation, tamoxifen pills will be provided (Arm 1 and 2). All participants will receive a calendar in order to record drug intake, ICR dates and weekly step counter where appropriate. The study interventions will last 6 months.

To maintain high compliance during the 6-month intervention phase (T2, T3 and T5) participants will receive phone calls in order to monitor and reinforce adherence to the proposed intervention, and to record possible adverse effects. At visit T1, T4 and T6 blood samples will be collected, anthropometric measures, study questionnaires will be collected. Stool will be collected at T1 and T6. Depending on local availability, a sub-sample of participants will be invited to do DEXA for adipose tissue evaluation.

Mammogram will be performed only in a subgroup of participants based on their age and center guideline, and can be within 6 or 12 months from baseline visit depending on disease status.

# 4. PARTICIPANT SELECTION

## 4.1 Inclusion Criteria

1. Women between 18 and 70 years old;
2. Healthy participants carriers of a germline pathogenic/likely pathogenetic variant (*BRCA1* or *BRCA2* or *PALB2*), **or**

> 5% Tyrer-Cuzick BC risk at 10 years, **or**

with previous diagnosis of intraepithelial neoplasia (surgery for ADH, LCIS, ER positive DCIS) within the last 3 years;

1. Ability to understand and the willingness to sign a written informed consent document.
2. ECOG performance status ≤1 (Karnofsky ≥70%; Appendix B);

5a. For high risk strata: A negative mammogram or any radiological image based on age and center protocol screening within 6 months before baseline visit;

5b. For IEN Strata: A negative mammogram within 12 months before baseline visit

6. A negative transvaginal ultrasound within 6 months before baseline visit.

## 4.2 Exclusion Criteria

1. Diagnosis of ER negative (<10%) DCIS, or history of breast invasive cancer in the previous 5 years;
2. Previous treatment with SERMs or any other hormonal treatment for breast neoplasms;
3. BMI < 18.5 Kg/m^2^ **and/or** Malnutrition Universal Screening Tool (MUST) score ≥2 **and/or** any current or past eating disorders;
4. Any diagnosis of invasive neoplasia, except non-melanoma skin cancer, in the previous 5 years;
5. Any tamoxifen contraindications (abnormal liver function, previous ischemic heart disease, endometrial disorder, previous deep venous thrombosis, history of pulmonary embolus, current or suspected glaucoma, retinopathy and cataract);
6. Current use of warfarin or other anticoagulant drugs
7. Bilateral mastectomy;
8. Pregnancy or desire to become pregnant in the subsequent 9 months after treatment cessation;
9. Diabetes or any other clinical condition that at the investigator’s discretion contraindicates the proposed intervention.
10. No hormonal contraception is allowed during study intervention. Non-hormonal methods will be advised for women of childbearing potential (WOCBP),.

Women must remain abstinent (refrain from heterosexual intercourse) or use non hormonal contraceptive methods with a failure rate of <1% per year during the intervention period and, specifically, from 9 months after the final dose of tamoxifen. A woman is considered to be WOCBP if she is post-menarchal, has not reached a postmenopausal state (>12 continuous months of amenorrhea with no identified cause other than menopause), and is not permanently infertile due to surgery (i.e., removal of ovaries, fallopian tubes, and/or uterus) or other causes determined by the investigator (e.g. Műllerian agenesis). Examples of non-hormonal contraceptive methods with a failure rate of <1% per year include bilateral tubal ligation, male sterilization, and copper intrauterine devices.

# 5. Interventions and AGENT ADMINISTRATION

## 5.1 Interventions and arms description

Potentially eligible participants will be contacted by a member of the investigator’s team who will explain the study and present lifestyle goals according to national and international recommendations (WCRF. 2018; CREA, 2019 https://www.crea.gov.it/web/alimenti-e-nutrizione/-/linee-guida-per-una-sana-alimentazione-2018). Briefly, WCRF recommendations (WCRF, 2018) specify that individuals should maintain body weight in the normal range, eat vegetables every day, limit daily consumption of energy-dense foods, sugary drinks, red meat, and alcohol, and to be physically active as part of everyday life; to be moderately physically active, equivalent to brisk walking for at least 30 min a day; as fitness improves, to aim for ≥60 min of moderate or ≥30 min of vigorous physical activity every day; and to limit sedentary habits such as watching television, reading a book, playing pc games.

Women expressing their interest in joining the study and after signing the informed consent will be scheduled for baseline visit. After confirming all inclusion and exclusion criteria they will be randomized to one of the following arms:

*Arm 1*

Low dose Tamoxifen (LDT)

Agent: Tamoxifen. Doses: 10 mg every other day

*Arm 2*

Low dose Tamoxifen (LDT) + Intermittent Caloric Restriction (ICR)

Agent: Tamoxifen. Doses: 10 mg every other day

*Arm 3*

Lifestyle intervention (LI) making use of a step counter device

*Arm 4*

Lifestyle intervention (LI) making use of a step counter device + Intermittent Caloric Restriction (ICR).

Duration of intervention: 6 months

*Description of Interventions:*

Low dose Tamoxifen administration

Participants will receive a 7-month drug supply. The participant will be advised to take one tablet (10 mg) every other day (e.g. every odd days) at dinner, always at approximately 8 PM.

Details on treatment administration (e.g., dose and timing) should be noted in the source documents and on the eCRF.

Intermittent Caloric Restriction

We will propose the so-called “5:2 diet,” with 5 days/week at regular energy intake and 2 days/week at an ∼75% energy deficit (the diet will be restricted at 500-700 Kcal per day, corresponding to a 75% reduction compared to normal intake). Detailed personalized meal plans will be created for each participant, in which portion sizes are reported and possible choices for meal components arranged by food groups to meet the target caloric amount foresee. Participants will receive a leaflet containing dietary advice and physical activity recommendations to be followed for the study period. During the 2 days of energy restriction, a daily intake of a minimum of 2 L of liquids (water, tea or infusions) will be strongly recommended to avoid dehydration and side effects of fasting such as hunger and lightheadedness, increased cold sensitivity, weakness and headaches. Trained dietitians/nutritionists will provide personal nutrition consultations at baseline and during the study period. The intervention will be monitored and reinforced by phone call at month 1, 2 and 5, and to monitor possible side effects. The first personal consultation within the first month will be used to motivate participants, to monitor compliance, and to consolidate knowledge about dietary programs proposed. Participants will be instructed to complete a fasting diary to monitor the frequency of energy-restricted days. During the 2 days of ICR, women will be advised to avoid tiring and particularly intense activities. If women are used to practice sport, it will be suggested to walk or practice yoga, meditation and light pilates sessions.

Lifestyle intervention

Participants will receive personalized advice on healthy lifestyle and the use of a step counter device. These devices tend to encourage both increased physical activity and decreased sedentary time. They are able to record total steps taken, total distance traveled, total time active during the day and the greatest length of time of consistent movements done during the day. A goal of "10.000 steps" per day has been widely promoted and advocated as a strategy for increasing physical activity among able adults. Participants will be asked to wear the step counter during the 6-month intervention period. They could use a smartphone or a tablet application to get feedback on their activity and sedentary time.

Training sessions for nutritionists/dietitians involved in the intervention (lifestyle and caloric restriction) will be organized by IEO as a coordinating center, in order to standardize nutritional counseling and interventions.

## 5.2 Contraindications

Eligible participants should not have specific contraindication to tamoxifen, considering also the short period of treatment. Any pre-existing condition that may impact on participant quality of life should be carefully evaluated before enrolling the participants. Furthermore, medications that may impact participant safety or scientific integrity of the study are non-allowed, such as strong CYP 2D6 inhibitors (*e.g*., paroxetine, fluoxetine or St. John’s wort).

Women with documented history of diabetes, current or past eating disorders, uncontrolled intercurrent illness will be excluded.

## 5.3 Concomitant Medications

All medications (prescription and over-the-counter), vitamin and mineral supplements, and/or herbs taken by the participant will be documented on the concomitant medication CRF and will include: 1) start and stop date, dose and route of administration, and indication. Medications taken for medical procedure (*e.g*., biopsy) should also be included.

##

## 5.3.1 Permitted and prohibited medications

The use of tamoxifen in patients receiving dicumarol-type anticoagulant therapy may significantly increase anticoagulant activity; this association must be avoided.

Tamoxifen combination with cytotoxic drugs may increase risk of thromboembolic events episodes this association must be avoided.

Tamoxifen metabolism uses mainly the CYP3A4 and CYP2D6 enzyme. Concomitant medications that interact with these enzymes may result in reduced tamoxifen efficacy. In particular, the combination of tamoxifen and certain SSRI antidepressants or other inhibitor of CYP2D6 (e.g., paroxetine, fluoxetine, quinidine, cinacalcet, or bupropion) should be avoided, since a reduction of tamoxifen efficacy cannot be excluded.

Potentially, any other drug is permitted: participants can take over-the-counter drugs; for drugs prescriptions, participants are asked to inform the investigational staff before treatment initiation.

## 5.4 Intervention Modification

Participants will be asked to maintain the assigned intervention throughout the treatment period. In particular, for the tamoxifen arms due to the short time of treatment no dose modification will be applied.

Toxicity will be evaluated using the NCI terminology criteria (CTCAE version 5.0).

If grade 1 or 2 toxicity occurs, the participant will be maintained on treatment irrespective of attribution to the study drug. However, treatment discontinuation should be planned if a persistent/recurrent and intolerable Grade 2 toxicity occurs.

In case of grade 3 toxicity unrelated, unlikely or possibly related to study treatment, participant may remain on treatment as per physician judgment. Women who experience other grade 3 or more severe adverse events will be removed from intervention.

| Toxicity | Attribution to study arm | | | | |
| --- | --- | --- | --- | --- | --- |
| Grade | Unrelated | Unlikely | Possible | Probable | Definite |
| 1 | C | C | C | C | C |
| 2 | C | C | C | C | C |
| 3 | C^1^ | C^1^ | C^1^ | W | W |
| 4 | W | W | W | W | W |

C = Continue intervention; C^1^ = Continue intervention as per physician judgment; W = Intervention withdrawal.

## 5.5 Adherence/Compliance

To be compliant, a participant has to take ≥ 75% of the scheduled pills and/or follow 75% of the expected days of intermittent caloric restriction as per assigned arm. We propose the use of multiple methods of adherence monitoring: subject self-reporting, diary completion and tablets count.

*Diary completion*: All participants will receive at each visit a calendar to facilitate tracking and recording of protocol treatment (Appendix C). Each participant will be asked to mark or write Yes/No in the corresponding day of the diary. Based on treatment assignment, participants are asked to fill the diary and some additional space will be left for participant’s notes.

A total of 120 pills will be supplied in Arms 1 and 2. Each participant will be asked to return all full and empty blisters.

Should the tablets not be returned, compliance will be calculated from the diary only.

Participants on Arms 2 and 4 will be asked to mark yes/no in the corresponding dates of ICR.

Participants on Arms 3 and 4 will be asked to record the step count number on a weekly basis.

An according-to-protocol subgroup analysis will be restricted to women who are “compliant”, such as participants who have taken ≥ 75% of the pills, for those randomized to tamoxifen and ≥ 75% of the days with the respected caloric restriction, by the reported diary

# 6. PHARMACEUTICAL INFORMATION

## 6.1 Name of experimental drug

Generic name: Tamoxifen

Chemical name: (z)-2-[4-(1,2-Dyphenyl-1-butenyl)phenoxy]-N,N-dymethylethanamine

Product classification: non-steroidal estrogen antagonist

CAS Registry No: 10540-29-1

Molecular formula: C26H24NO

Molecular weight: 371.53

Key chemical characteristics: fine, white odorless crystalline powder, mp 140-142°C. Slightly soluble in water, soluble in methanol, ethanol, acetone. Hygroscopic at high relative humidities.

Tamoxifen tablets: tamoxifen citrate, lactose, maize starch, hypromellose, magnesium stearate, sodium glycolate starch, macrogol 300, titanium dioxide.

## 6.2 Reported Adverse Events and Potential Risks

The most commonly reported adverse events (AEs) by early breast cancer patients treated with tamoxifen were (in order of frequency): hot flushes, sweating increased, gynecological disorders, insomnia, and dizziness., musculoskeletal disorders (in particular cramps), peripheral edema, pruritus, reduction in bone mineral density in premenopausal women. At hematological/biochemistry level, leucopenia, lymphocyte count decreased, and liver enzymes increase. A detailed list of adverse events and their frequencies is reported in the Tamoxifen SmPC given at 20 mg/day dose.

## 6.3 Availability

Tamoxifen tablets will be purchased, packed and labeled by the

IEO Pharmacy

Via Ripamonti 435

20141 Milan, Italy

Director: Emanuela Omodeo-Salè

Each box will report Study number, PI name, and expiring date.

## 6.4 Agent Distribution

The IEO Pharmacy department will be responsible for investigational drug distribution. Dr. Bonanni, as PI of the study and the local Site Investigators or their designees will be responsible for drug administration. They can delegate other study investigators (MDs and research nurses) to distribute the agent on their behalf.

## 6.5 Agent Accountability

The Investigator, or a responsible party designated by the Investigator, must maintain an updated record of the inventory and disposition of all agents received from IEO pharmacy. The Investigator is required to maintain adequate records of receipt, dispensing and final disposition of study agent. This responsibility has been delegated to the local pharmacist*.* Include on receipt record from whom the agent was received and to whom study agent was shipped, date, quantity and batch or lot number. On dispensing record, note quantities and dates study agent was dispensed to and returned by each participant.

## 6.6 Packaging and Labeling

Commercially available 10 mg Tamoxifen will be re-packaged and labeled according to EU Regulation 536-2014 article 61 par.5. Drug boxes will be distributed by IEO Pharmacy to each Institutional Pharmacy.

Tablets will be packaged in blisters. A two-part label will be used, 1 tear-off remains on the box, 1 tear-off on the source document (Appendix D).

The fixed part of the label will remain attached to the box and will identify the following:

Tamoxifen 10 mg: 120 Tablets

Expiration Date: XX-XX-XXXX

Study Number:

Patient ID: _________________________

Dispensed Date: _____________________

Take one tablet every other day at the same time.

Store at Controlled Room temperature 15°C - 25°C

Keep Out of Reach of Children

CAUTION: DRUG – LIMITED TO INVESTIGATIONAL USE ONLY

## 6.7 Storage

Study drug will be stored in a secure location at controlled room temperature, with excursions permitted between l5°-25°C at each Institutional Pharmacy*.*

## 6.8 Agent Destruction/Disposal

At the completion of investigation, all unused study agent will be returned and disposed by the local Institute Pharmacy.

# 7. CLINICAL EVALUATIONS AND PROCEDURES

## 7.1 Schedule of Events

The possibility of taking part to this trial will be presented to all the eligible mutation carriers followed in our clinics, or to women with a 10 years BC risk >5% by Tyrer-Cuzick model, or patients with a diagnosed ER-positive DCIS or any LCIS or ADH after standard surgery. The schedule of events at each time point is reported below.

**SCHEDULE OF EVENTS**

| Evaluation/ Procedure | T0  Screening Visit  Days -30-1 | T1  Baseline Visit/Randomization  Day 0 | T2  Phone call/ teleconsulting  (within 1 month) | T3  Phone call/ teleconsulting  (2 months) | T4  Visit  (3 months) | T5  Phone call/ teleconsulting  (5 months) | T6  Final Visit  (6 months) |
| --- | --- | --- | --- | --- | --- | --- | --- |
| Informed Consent | X |  |  |  |  |  |  |
| Stool Collection kit | X |  |  |  | X |  |  |
| Assess eligibility | X | X |  |  |  |  |  |
| Medical history |  | X |  |  |  |  |  |
| Physical exam/ anthropometric measures and body composition (BIVA) |  | X |  |  | X |  | X |
| Tobacco/alcohol use assessment |  | X |  |  |  |  |  |
| Fasting blood collection^a-b^ |  | X^b^ |  |  | X |  | X^b^ |
| Gravindex |  | X* |  |  |  |  |  |
| Stool collection |  | X |  |  |  |  | X |
| Mammography (according to Institutional guidelines)^c^ |  | X |  |  |  |  | X |
| Body fat composition (DEXA)^d^ |  | X |  |  |  |  | X |
| Concomitant medications |  | X |  |  | X |  | X |
| Baseline Symptoms |  | X |  |  |  |  |  |
| Randomization |  | X |  |  |  |  |  |
| Nutrition counseling |  | Arms 2, 4 only | Arms 2, 4 only | Arms 2, 4 only | Arms 2, 4 only | Arms 2, 4 only |  |
| Physical activity counseling |  | Arms 3, 4 only | Arms 3, 4 only | Arms 3, 4 only | Arms 3, 4 only | Arms 3, 4 only |  |
| Tamoxifen safety |  |  | Arms 1, 2 only | Arms 1, 2 only | Arms 1, 2 only | Arms 1, 2 only | Arm 1, 2 only |
| WCRF recommendations | X | X |  |  |  |  |  |
| Collect study agent |  |  |  |  |  |  | X |
| Compliance |  |  | X^g^ | X^g^ | X^e^ | X^g^ | X^e^ |
| Review diary |  |  |  |  | X |  | X |
| Adverse events |  |  | X | X | X | X | X |
| Study Questionnaires: QoL, DIET and IPAQ | X^f^ | X |  |  | X |  | X |

* In women of childbearing potential only

a. Glycemia will be measured locally and blood specimen will be collected for biomarkers as per Manual of procedure

b. Additional blood collection in dedicated tubes for RNA extraction as per Manual of procedure.

c. Mammogram will be performed only in a subgroup of participants based on their age and center guideline, and can be within 6 or 12 months from baseline visit depending on disease status.

d. In a subgroup of participants only, depending on local availability.

e. Revision of diary completion only. Unused and/or empty blisters will be returned at month 6 visit arms 1,2

f. 3-day food records only

g. Compliance with Intermittent Caloric Restriction

## 7.2 Pre-study screening visit

During the screening visit, the health care professional will illustrate the aim and the trial procedures to the candidate and eligibility will be assessed. If the woman wishes to consider to participate, she will be asked to sign the informed consent. We will provide her the stool collection kit and instruct her about baseline sample collection. The baseline visit will be scheduled within 30 days.

Information on dietary habits will be obtained to facilitate personalized consultation sessions.

## 7.3 Baseline

Baseline evaluations are:

Fasting glycemia and blood collection for circulating biomarkers; stool sample collection. Date of last menses will be recorded. Physical exam and medical history, vital signs, alcohol and tobacco assessment, anthropometric measurement (height, weight, waist measurement, hip measurement), body composition (BIVA), quality of life assessment; food consumption and level of physical activity enquired through self-administered questionnaires as explained below. Gravindex test will be obtained in women of childbearing potential.

The use of concomitant medications and any baseline symptom will be recorded.

At baseline, participants will be instructed about the importance of drug compliance, and lifestyle recommendation/instruction based on the randomization arm.

Participants will be provided and instructed on how to fill-up the calendar that will be dated and signed.

Participants will be informed that a pill count will be conducted on their study medication use so that their study medication blisters with remaining tablets must be returned at the clinic visit.

**STUDY QUESTIONNAIRES**

Quality of life will be assessed using the self-administered MenQoL questionnaire (Mapi Research Trust, France) during all visits. This tool is based on 29 items divided into four domains (vasomotor, physical, psychosocial, and sexual). Each item is scored from 1 to 8: 1 means no symptom, 2 indicates presence of the symptoms but not bothersome, 3 to 8 mean an increasing grade of discomfort. The vasomotor domain includes three items to investigate hot flashes and sweating. In the physical domain, there are 16 items covering general symptoms, skin, gastrointestinal, sleeping problems, and urinary symptoms. The psychosocial domain includes seven items, evaluating states of anxiousness, memory, and loneliness. The sexual domain has three items: sexual desire, vaginal dryness, and avoiding intimacy (see Appendix E).

**FOOD CONSUMPTION**

Food consumption will be measured at all visits using a short, self-administered questionnaire^51^ recently developed to assess adherence to the Mediterranean diet in the Italian population. In this contest, it will be used to record daily or weekly intake of the main food groups over the previous months and the change over the study (see Appendix E).

**INTERNATIONAL PHYSICAL ACTIVITY QUESTIONNAIRE**

Physical activity will be measured with the IPA Questionnaire (IPAQ) at all visits. This questionnaire consists of questions that record the frequency and duration of mild, moderate, and strenuous exercise performed during free time in the previous 7 days. It measures physical activity and inactivity.

It is a validated self-reported measure of exercise that has been reliably used in previous studies^52^. The total hours per week spent in each activity will be multiplied by the estimated metabolic cost of each activity (metabolic equivalent (MET) value) as determined from the Compendium of Physical Activities^53^ (see appendix).

**BODY COMPOSITION MEASUREMENTS**

Body composition will be assessed at T1, T4 and T6, using bioelectrical impedance vector analysis (BIVA) (Nutrilab device, AKERN Srl – Italy). BIVA is an accurate method for a quick measurement of body compartments^54, 55^. The direct analysis of the two components of the impedance vector (Z), resistance (R, Ohm) and reactance (Xc, Ohm), allows a semiquantitative evaluation of body composition in terms of body cell mass and hydration status. Data for total body water (TBW), body cell mass (BCM), extracellular water (ECW), fat-free mass (FFM), fat mass (FM) and percentage fat mass (% FM) will be available for all participants and will be used for identifying changes of fat and fat-free mass over the study period.

Depending on local availability, in a sub-sample of women, body composition will be quantified via dual-energy X-ray absorptiometry (DEXA) at T1 and T6. DEXA is considered the gold standard for total body composition analysis^56^, but it is a medical exam and not a regularly used for body composition assessment. In this study, DEXA will be used to measure tissue absorption of high- and low-energy X-ray beams that pass through the participant in a supine position. Those measurements provide a multicompartment assessment, including FM, FFM, and bone mineral density. The DEXA device automatically calculates the body composition of each participant.

## 7.4 Evaluation During Study Intervention

A phone contact or tele-consulting will be performed at T2, T3, T5 to motivate and support adherence to ICR, LI, and to monitor tamoxifen toxicity.

## 7.5 Three-month visit

Fasting blood will be drawn for glycemia and blood circulating biomarkers. A stool kit collection will be provided to the participant, to be collected at the final study visit. Date of last menses will be recorded.

Physical exam, vital signs, anthropometric measurement including BIVA, quality of life, food habit and physical activity will be assessed by self-administered questionnaires.

**7.6 Final visit**

Fasting blood drawing for glycemia and circulating biomarkers; stool sample collection. Date of last menses will be recorded

At the final visit, participants will undergo physical exam, vital signs, mammogram if indicate, and anthropometric measurements including BIVA.

The use of concomitant medications, adverse events, quality of life, food habit and physical activity will be assessed. Study agent will be collected and compliance will be evaluated by pills counts and review of agent diary.

## 7.7 Off-Agent Criteria

Participants may interrupt assigned intervention or may stop taking LDT for the following reasons: completed the protocol-prescribed intervention, adverse event or serious adverse event, inadequate agent supply, non-compliance, concomitant medications, medical contraindications, withdrawn consent, positive serum pregnancy test. Participants will continue to be followed, if possible, for safety reasons and in order to collect endpoint data according to the schedule of events. Participants will not be replaced*.*

## 7.8 Off-Study Criteria

Participants may go ‘off-study’ for the following reasons: the protocol intervention and any protocol-required follow-up period is completed, adverse event/serious adverse event, lost to follow-up, non-compliance, concomitant medication, medical contraindication, withdrawn consent, death, determination of ineligibility (including screen failure)*.*

## 7.9 Study termination

Primary completion date will be the date that the last participant will undergo the final visit for the purpose of final collection of data for the primary outcome.

Study completion date will be the final date in which all secondary endpoints will be assessed.

## 7.10 Study and site closure

The Sponsor reserves the right to close the study site or terminate the study at any time for any reason at the sole discretion of the Sponsor. Study sites will be closed upon study completion. A study site is considered closed when all required documents and study supplies have been collected and a site closure visit has been performed. The investigator may initiate site closure at any time, provided there is reasonable cause and sufficient notice is given in advance of the intended termination.

Reasons for the early closure of a study site by the Sponsor or investigator may include, but are not limited to, the following:

• Failure of the investigator to comply with the protocol, the requirements of the IRB/EC or local health authorities, the Sponsor's procedures, or the ICH Guideline for Good Clinical Practice

• Inadequate recruitment of participants by the investigator

• Discontinuation of further study treatment development. If the study is prematurely terminated or suspended, the Sponsor shall promptly inform the investigators, the EC and the health authorities of the reason for termination or suspension. The investigator shall promptly inform the participants and should ensure appropriate participant therapy and/or follow-up.

# 8. CRITERIA FOR EVALUATION AND ENDPOINT DEFINITION

## 8.1 Primary Endpoint

The main endpoint of the study will be serum concentrations (nmol/L) of SHBG after 6 months of intervention. We expect to obtain a difference of 15 points comparing the two low dose tamoxifen arms with the two other arms that include lifestyle intervention (step counter), with or without intermittent-caloric restriction^57, 58^. The measurement will be centralized at the Division of Cancer Prevention and Genetics at the IEO.

## 8.2 Secondary Endpoints

-Change in time of SHBG, insulin, lipid profile, glucose (HOMA-index), IGF-I bioavailability (IGF-I, IGF binding proteins: IGFBP-1, -2 and -3), and Adiponectin/Leptin ratio.

-Change in quality of life (QoL) measured by MenQoL questionnaire.

-Body Mass Index, hip and waist circumference variations

-Body composition/fat distribution changes (measured by BIVA),

-Safety and toxicity (in particular for the combined intervention).

-Evaluation of differences in microbioma changes in time by arms (Tam LD vs lifestyle intervention).

- Immune-modulation by expression of inflammation cell signaling genes

In a subgroup of participants, MD and DEXA changes will be assessed.

Fasting glucose will be determined locally at each visit by the methodology adopted by each center; all other biomarkers will be measured as described in section 9.1.

In addition, quality of life will be assessed by a self-administered Quality of Life questionnaire (MenQoL); moreover, safety and toxicity will be evaluated at the clinical visit according to the Common Terminology Criteria for Adverse Events v5.0 (CTCAE).

Body composition will be evaluated by the raw bioelectrical impedance parameters by the bioelectrical impedance vector analysis (BIVA). The specific BIVA approach represents a suitable method for evaluating FM% and its changes following a lifestyle intervention program. In particular, vector displacements towards the lower pole of an R-Xc graph were found to reflect decreases in FM% after an intervention program aimed to reduce body fat. BIVA for body composition analysis will be provided to each study center.

Depending on local availability and/or women acceptance, body composition will be assessed also by patient’s DEXA according to bi-compartmental (fat mass and fat-free mass) or three-compartmental (fat mass, lean soft tissue, and bone mineral content) models. Participants will be invited to perform a DEXA at baseline and after 6 months.

Moreover, mammography breast density will be performed in a subgroup of participants (not all the participants will undergo mammography due to younger age).; the volumetric breast density from digital mammograms will be evaluated centrally (at the IEO) by the use of the Volpara software.

# 9. Collection and Handling Procedures

We will collect and process serum, whole blood and stool for evaluation of biomarkers. Specific instructions about specimen handling and storage will be provided in a separate Manual of Operations and Procedures. Storage of serum and blood samples will be centralized at the Laboratory of the Division of Cancer Prevention and Genetics, IEO, Milan. They will provide kits for sample collection and check sample identification at arrival and coordinate the shipment of samples to the specific laboratories involved in biomarker measurements.

## 9.1. Blood

Blood samples for serum biomarkers will be collected at baseline, three and six months, whole blood (EDTA) will be collected only at baseline, whole blood for RNA samples will be collected at baseline and six months.

Blood samples for biomarkers will be drawn under fasting condition (at least 6 hours) preferably between 8 a.m. and 10 a.m. at each visit. A total of 25 ml of blood (5 x 5mL) will be collected into vacuum blood collection tubes containing beads coated with clotting activator for serum separation to be employed for circulating biomarker analysis. Based on specific tubes adopted locally, reference to the manufacture instructions will be followed. blood will be allowed to clot at room temperature for 30 minutes. Then, it will be spun in centrifuge for 10 minutes. Sarstedt Serum Monovettes tubes require 10 min centrifugation at 2000 x g at room temperature, while BD Vacutainer® SST™ and PST™ gel tubes should be spun at a speed of 1300 x g. Swing-out buckets best option for both systems. After centrifugation, the yellow top layer, which corresponds to serum, is pipetted using the disposable transfer pipettes in 6 even aliquots (about 2 mL each) into the polypropylene cryotubes (Thermo Fisher Scientific), specifically labeled according to the Instructions in the Manual of Operations and Procedures. Tubes are tightly capped and stored in a dedicated – 80°C freezer equipped with a temperature control and temperature log chart or an alarm monitoring system per institutional standards.

Moreover, **3 ml whole blood** will be collected in tubes containing EDTA-K2 as anti-coagulant agent. The tube is gently mixed and blood is pipetted using disposable transfer pipettes in 2 even aliquots (about 1.5 mL each) into the polypropylene cryotubes (Thermo Fisher Scientific), specifically labeled according to the Instructions in the Manual of Operations and Procedures. Tubes are tightly capped and stored in a dedicated –80°C freezer equipped with a temperature control and temperature log chart or an alarm monitoring system per institutional standards.

Additionally, **2.5 mL whole blood** will be drawn into PAXgene® Blood RNA Tubes, for immediate stabilization of intracellular RNA, and stored at -20°C to -80°C.

**Specimen collection kits** for storage of serum and blood samples will be provided by the Central Laboratory, at the Division of Cancer Prevention and Genetics, European Institute of Oncology in Milan, Italy (See the Instructions in the Manual of Operations and Procedures for requesting kits). Briefly, they will provide labels and polypropylene cryotubes and rack (Thermo Fisher Scientific) and instructions for specimen handling, processing, labeling, tracking and storage.

Each local site must be equipped with a -80°C (range -70 °C to -80 °C) freezer provided with temperature control 24 hours a day, 7 days a week and temperature log charts or an alarm monitoring system, better if electronic. The center should also be equipped with a back-up freezer.

## 9.2 Stool

Fecal samples will be collected for microbiome analysis at baseline and final visit. Specimens will be collected by patients at home and transported in a collection tube prefilled with preservative liquid, which stabilizes nucleic acids preventing them from being degraded up to 6-8 weeks at room temperature.

Specimen collection kits for storage of fecal samples will be provided by the European Institute of Oncology in Milan.

## 9.3 Biomarkers Methods

**Sex hormone binding globulin (SHBG)**

Serum concentrations of SHBG will be determined by a chemiluminescent immunoassay designed for the IDS-iSYS Multi-Discipline Automated System (Immunodiagnostic Systems Limited, United Kingdom). Lower Limit of Quantitation is 0.30 nmol/L.

**Insulin and lipids**

Serum concentrations of insulin will be determined by a chemiluminescent microparticle immunoassay (CMIA) on the ALINITY *i* System (Abbott Laboratories, Weisbaden, Germany). This assay shows very good agreement with our previously adopted platforms and the assay performance in terms of reproducibility were improved. The sensitivity of the method is ≤ 1.0 μU/mL, and inter-assay coefficient of variation are below 2% at 3 different control levels (low, median, high) produced by Abbott. Inter-assay coefficient of variation of our in-house prepared serum pool (mean: 4.9 μU/mL) is 3.9%. Additionally, we will measure total, LDL, HDL cholesterol and triglycerides levels by the same method.

**HOMA-index**

We will calculate the HOMA index, i.e. [fasting insulinemia (mU/L) x glycemia (mmol/L)]/22.5, to be applied as a surrogate index of insulin resistance.

**Adiponectin and leptin**

Serum adiponectin, leptin and cytokines (IL-6 TNF-alpha) will be measured using an automated platform for immunoassays (ELLA system, ProteinSimple). ELLA is a platform based on a microfluidic technology, that allows to perform automated immunoassays without manual steps in 72 minutes. The inter-assay CVs for adiponectin QCs and our internal control are 4.98% and 6.62%. The inter-assay CVs for leptin QCs and our internal control are 6.77% and 5.35%. The inter-assay CVs of our pooled serum for IL-6 and TNF-alpha were 5.4% (mean 1.26 pg/mL) and 4.6 % (mean 6.89 pg/mL), respectively

**Insulin like growth factor I (IGF-I) and IGF binding protein 3 (IGFBP-3)**

Serum concentrations of IGF-I and IGFBP-3 will be measured by means of commercially available assays, intended for the quantitative determination in human serum or plasma on the IDS-iSYS Multi-Discipline Automated System. Serum samples from the same participants obtained at different time-points will be run in batches in order to reduce analytical variability. Standards for good laboratory practice are applied. Monitoring of precision and reproducibility will be performed by processing commercially available control samples and internal in-house prepared pool of samples in each run.

**Insulin like growth factor binding protein 1 (IGFBP-1) and IGFBP-2**

IGFBP1 and IGFBP-2 will be measured by the Human ELISA (Enzyme-Linked Immunosorbent Assay) kit is an in vitro enzyme-linked immunosorbent assay purchased from Abcam Cambridge, UK.

**hsCRP (high sensitive C-reactive protein)**

We will measure serum concentrations of C-reactive protein by a high sensitivity turbidimetric method according to the manufacturer’s instructions (Roche Diagnostics, Mannheim, Germany). The test is designed for the automated instrument COBAS INTEGRA 800. Sensitivity for the hsCRP assay is 0.1 mg/L and the intra- and inter-assay coefficients of variation are expected to be 4.1% and 6.4%, respectively for a control sample of 0.423 mg/L.

**Immune Responses - Gene expression profile**

RNA will be extracted by Qiagen Kit, then subjected to reverse transcription reaction using RT2 first strand kit (Qiagen). Real-Time qPCR performed in triplicate using RT2 Profiler PCR Array (RT² Profiler- PCR Human Innate & Adaptive Immune Responses, Qiagen) for expression of inflammation cell signaling genes. Plates will be run on ViiA7 (Applied Biosystems 384 well blocks), according to standard PCR protocols and online software (http://pcrdataanalysis.sabiosciences.com/pcr/arrayanalysis.php, Qiagen). Cell signalling pathway using CellFate algorithm.

**Microbiome**

Microbial genomic DNA will be extracted from frozen samples using DNeasy PowerSoil Pro Kit (Qiagen) according to the manufacturer’s instructions, then DNA will be quantified using a Bioanalyzer (Agilent Technologies, CA) and the V3-V4 hypervariable regions of the bacterial 16S rRNA gene will be sequenced on a MiSeq platform (Illumina), 26 enabling taxonomic identification. The 250 bp 16S reads will be processed through QIIME2 (version 2019.7)^59^ as follows: (1) Following visualization of demultiplexed samples and the average quality across the reads, quality filtering, dereplicating, and chimera filtering will be performed using the DADA2^60^ plugin within QIIME2, setting the truncation length at 250 bp and the trimming by the length of the V3-V4 primer sequences (--p-trunc-len-f 250, --p-trunc-len-r 250, --p-trim-left-f 17, --p-trim-left-r 2, --p-trunc-q 2), and using consensus as the chimera filtering method; (2) a phylogenetic tree will be generated for downstream core diversity analyses using SATe’-enabled phylogenetic placement (SEPP)^61^, which first generates a reference tree—in this case using the SILVA 128 database^62^ then inserts 16S sequence fragments into the tree, thus achieving accuracy in phylogenetic tree reconstruction while retaining as much sub-OTU sequences as possible in the tree alpha and beta diversity core metrics will be determined using the qiime diversity command, with the rarefaction depth set to the minimum sequence read output across the samples, after which statistical group comparisons of alpha and beta diversity metrics will be performed, using Kruskal-Wallis for alpha diversity and PERMANOVA for beta diversity; taxonomy classification will be performed using the QIIME feature-classifier classify-sklearn feature, using a Naïve Bayes classifier trained on SILVA 132 99% OTUs full-length 16S rRNA sequences^62^, available from the QIIME2 website (https://docs.qiime2.org/2018.11/data-resources/). After filtering the feature count table for unassigned reads and setting a prevalence filter of >50% of the samples, the tables will be collapsed to each taxonomic level (kingdom, phylum, class, order, family, genus, species) and will be exported for further analysis in R. Differential abundance analysis will be performed on the raw counts table using DESeq2^63^, Statistical significance of log2 fold changes will be assessed using the default Wald test with Benjamin-Hochberg p-value correction in DESeq2. Cut-off for all significance tests was set at P < 0.05. Amplicon-based metagenomic approach represents one of the best strategies to obtain a largescale assessment of the taxonomic content of complex samples while containing costs.

## 9.4. Study specimen and procedure management

Planned analysis of circulating and other biomarkers will be determined by specialized laboratories/departments as outlined below:

| **Specimen (amount)** | **Laboratory** |
| --- | --- |

| **Serum: 6 aliquots (2 mL each) per time point**  For all circulating biomarkers  At baseline, 3 months, and 6 months (or study cessation) | Harriet Johansson, MSc, PhD  Division of Cancer Prevention and Genetics  European Institute of Oncology |
| --- | --- |
| **Stool: 1 aliquot per time point**  Microbiota analysis at baseline and 6 months  **Whole blood 1 aliquot per time point**  Immune-gene expression profile | Istituto Nazionale Tumori G. Pascale, Naples |
| **Mammograms***  Baseline and final images will be recorded to mammographic analysis | Enrico Cassano MD  Department of Breast Radiology,  European Institute of Oncology |

*Only in a subgroup of participants

## 9.5 Shipping Instructions

In order to keep shipping costs at an affordable level, Participating Centers are requested to store blood and stool samples at -80°C. Dry-ice shipment of serum and blood samples will be arranged by the Lab at the Division of Cancer Prevention and Genetics, European Institute of Oncology in Milan. Storage of serum and blood samples will be centralized at the same Laboratory. Dedicated lab staff will provide kits for sample collection, will be in charge of sample identification and database implementation at arrival and the will coordinate the shipment of samples to the specific laboratories involved in biomarker measurements (i.e., Istituto Nazionale Tumori IRCCS Fondazione G. Pascale Napoli).

Contact:

Division of Cancer Prevention and Genetics

European Institute of Oncology

Via Ripamonti 435

20141 Milan, Italy

Harriet Johansson

Phone: +39 02 94372654

E-mail address: [harriet.johansson@ieo.it](mailto:harriet.johansson@ieo.it)

# 9.6 Specimen Banking

If the participant gives her consent, leftover specimens from the laboratory work will be stored at ieo biobank for future research studies on breast cancer prevention. Contrarily, the specimens will be destroyed after study completion. The biobank will store the specimens until depleted, and anyhow no longer than 25 years from collection. The donor will be re-contacted to give a new consent for the future use of leftover samples. In the event of a future secondary use, the study will be evaluated by the ethic committee.

# 10. REPORTING ADVERSE EVENTS (AE)

DEFINITION: AE means any untoward medical occurrence associated with the use of a drug in humans, whether or not considered drug related. An AE can therefore be any unfavorable and unintended sign, symptom, or disease temporally associated with participation in a study, whether or not related to that participation. This includes all deaths that occur while a participant is on a study.

Please note that all abnormal clinical laboratory values that are determined to be of clinical significance based on a physician’s assessment are to be reported as AEs. Those labs determined to be of no clinical significance or of unknown clinical significance (per the physician’s assessment) should not be reported as AEs. Any lab value of unknown clinical significance should continue to be investigated/followed-up further for a final determination, if possible.

A list of AEs that have occurred or might occur (Reported Adverse Events and Potential Risks) can be found in §6.2, Pharmaceutical Information, as well as in the Summary of Product Characteristics.

## 10.1 Adverse Events

Reportable AEs

All AEs that occur after the informed consent is signed and baseline assessments are completed must be recorded on the AE CRF (paper and/or electronic) whether or not related to study agent.

Cases of overdose, medication error, drug abuse, or drug misuse, along with any associated adverse events, should be reported.

AE Data Elements:

The following data elements are required for adverse event reporting.

- AE verbatim term
- NCI Common Terminology Criteria for Adverse Events version 6.0 (CTCAE v 5.0) AE term (MedDRA lowest level term)
- CTCAE (MedDRA) System Organ Class (SOC)
- Event onset date and event ended date
- Treatment assignment code (TAC) at time of AE onset
- Severity grade
- Attribution to study agent (relatedness)
- Whether or not the event was reported as a serious adverse event (SAE)
- Whether or not the subject dropped due to the event
- Outcome of the event

Severity of AEs

Identify the AE using the CTCAE v 5.0. The CTCAE provides descriptive terminology (MedDRA lowest level term) and a grading scale for each AE listed. A copy of the CTCAE can be found at:

https://ctep.cancer.gov/protocolDevelopment/electronic_applications/docs/CTCAE_v5_Quick_Reference_5x7.pdf

AEs will be assessed according to the grade associated with the CTCAE term. AEs that do not have a corresponding CTCAE term will be assessed according to the general guidelines for grading used in the CTCAE v5.0 as stated below.

**CTCAE v5.0 general severity guidelines:**

| Grade | Severity | Description |
| --- | --- | --- |
| 1 | Mild | Mild; asymptomatic or mild symptoms; clinical or diagnostic observations only; intervention not indicated. |
| 2 | Moderate | Moderate; minimal, local or noninvasive intervention indicated;  limiting age-appropriate instrumental activities of daily living (ADL)*. |
| 3 | Severe | Severe or medically significant but not immediately life-threatening;  hospitalization or prolongation of hospitalization indicated; disabling; limiting self-care ADL**. |
| 4 | Life-threatening | Life-threatening consequences; urgent intervention indicated. |
| 5 | Fatal | Death related to AE. |

* **ADL**

*Instrumental ADL refers to preparing meals, shopping for groceries or clothes, using the telephone, managing money, *etc*.

**Self-care ADL refers to bathing, dressing and undressing, feeding self, using the toilet, taking medications, and not bedridden.

Assessment of relationship of AE to intervention

The possibility that the adverse event is related to intervention will be classified as one of the following: not related, unlikely, possible, probable, definite.

Adverse events potentially related to tamoxifen treatment have been described in section 6.2 and are available in the SmPC of tamoxifen.

Fasting and intermittent fasting have been reported to cause some adverse events such as feeling cold, lack of energy, and occasional dizziness, nausea, insomnia, syncope, falls, migraine headache, weakness that limits daily activities, and excessive hunger pangs^64, 65^. We do not foresee any serious adverse event. However, if participants will experience an adverse event, this will be reported immediately to the attention of the clinical staff. All participants will be monitored to prevent any possible deficiency, dehydration or inadequate nutrient and/or caloric intake by means of an evaluation of their dietary intake. Participants will also be monitored for injuries or problems associated with the increased physical activity.

Participants are invited to contact the clinical sites to report any AE for clinical evaluation and management.

Follow-up of AEs

All AEs, including lab abnormalities that in the opinion of the investigator are clinically significant, will be followed according to good medical practices and documented as such.

## 10.2 Serious Adverse Events

Definition of SAE

Serious adverse event (SAE) is defined as one of the following:

Is fatal or life-threatening

Results in persistent or significant disability/incapacity

Constitutes a congenital anomaly/birth defect

Is medically significant, i.e., defined as an event that jeopardizes the patient or may require medical or surgical intervention to prevent one of the outcomes listed above

Requires inpatient hospitalization or prolongation of existing hospitalization.

Note that hospitalizations for the following reasons should not be reported as serious adverse events:

Routine treatment or monitoring of the studied indication, not associated with any deterioration in condition (i.e. to perform study related assessments)

Elective or pre-planned treatment for a pre-existing condition that is unrelated to the indication under study and has not worsened since signing the informed consent

Social reasons and respite care in the absence of any deterioration in the patient’s general condition

Note that treatment on an emergency outpatient basis that does not result in hospital admission and involves an event not fulfilling any of the definitions of a SAE given above is not a serious adverse event

Reporting

To ensure patient safety every SAE, regardless of suspected causality, occurring after the patient has provided informed consent and until at least 30 days after the patient has stopped study treatment must be reported to the EIO Pharmacy within 24 hours of learning of its occurrence.

Any additional information for the SAE including complications, progression of the initial SAE, and recurrent episodes must be reported as follow-up to the original episode within 24 hours of the investigator receiving the follow-up information. A SAE occurring at a different time interval or otherwise considered completely unrelated to a previously reported one should be reported separately as a new event.

Any SAEs experienced after the 30-day safety evaluation follow-up period should only be reported to the EIO Pharmacy if the investigator suspects a causal relationship to the study treatment.

Information about all SAEs is collected and recorded on the Adverse Event Report Form; all applicable sections of the form must be completed to provide a clinically thorough report. The Investigator must assess and record the relationship of each SAE to each specific study treatment (if there is more than one study treatment), complete the AE Report Form in English, and submit the completed form within 24 hours to EIO Pharmacy.

Follow-up information is submitted in the same way as the original AE Report. Each reoccurrence, complication, or progression of the original event should be reported as a follow-up to that event regardless of when it occurs. The follow-up information should describe whether the event has resolved or continues, if and how it was treated, whether the blind was broken or not (if applicable), and whether the patient continued or withdrew from study participation.

If the SAE is not previously documented in the Investigator’s Brochure or Drug Package Insert (new occurrence) and is thought to be related to the study treatment, the Safety Committee of EIO may urgently require further information from the investigator for Health Authority reporting. Suspected Unexpected Serious Adverse Reactions (SUSARs) will be collected and reported to the Competent Authorities and relevant Ethics Committees in accordance with applicable national regulatory requirements.

Pregnancies

Should pregnancy occur, to ensure patient safety, each event must be reported to the EIO Pharmacy within 24 hours of learning of its occurrence.

The pregnancy should be followed up to determine outcome, including spontaneous or voluntary termination, details of the birth, and the presence or absence of any birth defects, congenital abnormalities, or maternal and/or newborn complications.

Pregnancy follow-up should be recorded on the same form and should include an assessment of the possible relationship to the study treatment any pregnancy outcome. Any AE experienced during pregnancy must be reported on the AE Report Form.

The Study Doctor will collect information such as progression of the pregnancy, the pregnancy outcome and the health of the baby. The collection of this information could last for up to 3 months following the birth of the child, and longer if required by the Safety Committee. Consent to report information regarding these pregnancy outcomes should be obtained from the mother.

**E-mail contact for SAE and SUSARs notification and information is:**

**info.farmacovigilanza@ieo.it**

**(Emanuela Omodeo Salè)**

Warnings and precautions

No evidence available at the time of the approval of this study protocol indicated that special warnings or precautions were appropriate, other than those noted in the provided Summary of Product Characteristics or in the IB. Additional safety information collected between IB updates will be communicated in the form of Investigator Notifications. This information will be included in the patient informed consent and should be discussed with the patient during the study as needed.

EIO Safety Committee (SC)

The SC will ensure transparent management of the safety information through investigator notifications and recommending modifications as circumstances require. Together with the clinical trial team, the SC may be involved in evaluations about the risk/benefit ratio based on new risks evidenced by SUSARs.

# 11. STUDY MONITORING

## 11.1 Data Management

We will conduct the trial according to the ICH Good Clinical Practice (GCP) guidelines. Keeping accurate and consistent records is essential to a cooperative study.

The IEO Data Management Office will be responsible of the study database development and data management. IEO Data Management Office, Direzione Scientifica, Istituto Europeo di Oncologia -Via Ripamonti, 435 - 20141 Milano - T 0257489938 F 0255210169 M 3356055650

Data for this study will be collected in a REDCap® (Research Electronic Data Capture) database. REDCap was developed specifically around HIPAA-Security guidelines. More information about the consortium and system security can be found at <http://www.projectredcap.org/>. REDCap is a secure web platform for building and managing online databases and surveys. REDCap's streamlined process for rapidly creating and designing projects offers a vast array of tools that can be tailored to virtually any data collection strategy. REDCap provides an intuitive user interface that streamlines project development and improves data entry through real-time validation rules (with automated data type and range checks). REDCap also provides easy data manipulation (with audit trails for reporting, monitoring and querying patient records) and an automated export mechanism to common statistical packages (SPSS, SAS, Stata, R/S-Plus). Investigators who have received appropriate institutional research approval (i.e., Institutional Review Board or Institutional Ethics Committee) will be given a web link with a survey where they can enter data about their specific patients. Guidelines about the data collection and to properly enter the data will be developed. The Promoter/Study Coordinator Centre is the legal owner of the collected data and has the right to manage it (including the data collected by the centers involved) in the case of a multi-site study, the sharing of data to any satellite centers is at the discretion of the Coordinator Centre under existing contractual agreements the regulation regarding protected health information (PHI) is also valid in Italy: it is therefore not possible to enter sensitive data of the subjects enrolled in the study or any other data (such as hospital codes/labels/SDO) that can lead to their identity; that's why when a subject is inserted into the platform, the system assigns her/him a unique identifier. The id/name-last name code decoding is the responsibility of the PI of each experimental center (and should not be shared with the Coordinator Center). Each experimental center will then be the only one to be able to decode the IDs assigned to the subjects managed by its center.

Investigators will access the medical records of their patients, enter required data into the database. The protected health information will not be reused or disclosed to any other person or entity, except as required by law, for authorized oversight of the research project. Future research, that is not defined in this protocol, wishing to access the REDcap database will need institutional review board/ethic review board approval before obtaining access to the REDcap database.

## 11.1.1 Data Protection

Information technology systems used to collect, process, and store study-related data are secured by technical and organizational security measures designed to protect such data against accidental or unlawful loss, alteration, or unauthorized disclosure or access. In the event of data security breach, appropriate mitigation measures will be implemented.

## 11.1.2 Protocol Deviations

The investigator should document and explain any protocol deviations. The investigator should promptly report any deviations that might have an impact on participant safety and data integrity to the Sponsor and to the IRB/EC in accordance with established IRB/EC policies and procedures. The Sponsor will review all protocol deviations and assess whether any represent a serious breach of Good Clinical Practice guidelines and require reporting to health authorities.

## 11.2 Registration/Randomization

**Identification of participants**

According to good clinical practice (GCP) guidelines and current legislation, patients have a right to privacy. Therefore, case report forms (CRF) or any other document related to the study will not contain subject names. Subjects will be identified by a unique code attributed at the moment of study inclusion.

The local data manager will maintain the internal registry for the identification of all subjects participating in this study. The registry will contain:

- name and surname of the participant;
- medical record number;
- screening ID
- unique identification code;
- date of birth;
- date of inclusion in the study/signed informed consent.
- reason of ineligibility or refusal

Additionally, all institutional measures for the safeguard of patient privacy will be implemented.

Screening:

All potentially eligible participant will be contacted to propose the study, to this patient will be assigned a temporary ID number and only to the one will be actually sign the inform consent will receive the unique identification code

Randomization:

Randomization will be performed using REDCAP platform.

Participants will be randomized to one of the four intervention arms once eligibility has been verified at the site level, eligibility has been confirmed by the site PI or his designee, eligibility CRF has been entered into the DB web application. Once eligibility has been verified and confirmed, the allocated arm will be generated by the database and assigned to the participant. Refer to Section 12.2 for details of randomization.

Appropriate CRFs must be completed for any participant who signs an informed consent. If a consented participant is a screen failure and deemed ineligible, the following CRFs must be completed: 1) Screening CRF 2) Registration CRF; 3) Randomization CRF with the eligibility box checked “no”, 4) the Inclusion and Exclusion CRFs showing why the participant is ineligible, 5) the Off-Study CRF, 6) the Concomitant Medication CRF and 7) the Verification CRF. If no Concomitant Medications were assessed by the time the participant is deemed ineligible, the “NONE” box will be checked. All participants who sign an informed consent must formally go off study. All participant registration information will be entered into DB.

## 11.3 Case Report Forms

Participant data will be collected using protocol-specific case report forms (CRF). The approved CRFs will be used to create the electronic CRF (e-CRF) screens in the REDCAP application. Site staff will enter data into the e-CRF.

## 11.4 Source Documents

Source documentation will include only those documents containing original forms of data, including clinic charts, shadow files, hospital charts, questionnaires and physician notes. Data recorded directly on the CRFs designated as source documents (i.e., no prior written or electronic record of data) will be considered source data. All other data recorded on the CRFs will not be considered source documentation. Self-reported questionnaires and diaries will be considered source data.

## 11.5 Record Retention

Clinical records for all participants, including CRFs, all source documentation (containing evidence to study eligibility, history and physical findings, laboratory data, results of consultations, *etc*.), as well as IRB records and other regulatory documentation will be retained by the Investigator in a secure storage facility. The records should be accessible for inspection and copying by authorized persons of the Italian Ministry of Health representative and Italian Agency of Drug (AIFA), and to study monitors.

In compliance with the ICH/GCP guidelines, the investigator/institution will maintain all eCRFs and all source documents that support the data collected from each subject, as well as all study documents as specified in ICH/GCP Section 8, Essential Documents for the Conduct of a Clinical Trial, and all study documents as specified by the applicable regulatory requirement(s).

The investigator/institution will take measures to prevent accidental or premature destruction of these documents. Essential documents must be retained until at least 25 years after the last approval of a marketing application in an ICH region and until there are no pending or contemplated marketing applications in an ICH region or until at least 15 years have elapsed since the formal discontinuation of clinical development of the investigational product. These documents will be retained for a longer period if required by the applicable regulatory requirements or by an agreement with the sponsor. It is the responsibility of the sponsor to inform the investigator/institution as to when these documents no longer need to be retained.

If the responsible investigator retires, relocates, or for other reasons withdraws from the responsibility of keeping the study records, custody must be transferred to a person who will accept the responsibility. The sponsor must be notified in writing of the name and address of the new custodian. Under no circumstance shall the investigator relocate or dispose of any study documents before having obtained written approval from the sponsor. If it becomes necessary for the sponsor or the appropriate regulatory authority to review any documentation relating to this study, the investigator must permit access to such reports

# 12. STATISTICAL CONSIDERATIONS

## 12.1 Statistical Plan

*Sample size considerations*

Main objective

Given the results found in our previous studies^18, 57^, we considered as main endpoint SHBG (nmol/L) post treatment, which has been shown to be associated with risk of first cancer or cancer recurrence. Taking into account a drop-out rate of 10% we will have to enroll a total of 200 subjects. In fact, a sample size of overall 180 patients (90 per arm) achieves 80% power to detect a difference of 15 points between the null hypothesis that means of SHBG (nmol/L) post treatment are in both arms 70 and the alternative hypothesis that the mean in the tamoxifen arm is 85, with an estimated group standard deviation of 35 and with a significance level (alpha) of 5% using a two-sided two-sample t-test.

Secondary objectives

In order to investigate the effect of caloric restriction on biomarkers, we will subdivide each arm in two further groups: with and without caloric restriction, leading to a 4 arms design (45 patients per arms). Adjusting for multiple testing will be carry out for all secondary objectives that have an explorative nature.

## 12.2 Randomization/Stratification

In order to keep as low as possible the imbalance in treatments a stratified blocked randomization strategy will be used considering only the relevant prognostic factors. Participants will be stratified according to center and diseases status (healthy high risk versus affected).

## 12.3 Statistical analysis

Subject characteristics, information on lifestyle risk factors, diet and serum biomarkers at baseline will be summarized with descriptive characteristics. Median and interquartile range for continuous variables and absolute and relative frequencies for categorical variables will be presented by arms. Kruskal-Wallis test and Chi-square test (Fisher exact test for sparse data) will be used to assess differences at baseline between arms. Comparisons by affected versus high risk subjects of baseline serum biomarkers will be also carried out using Wilcoxon sum-rank test. We will employ linear regression models to investigate the associations between Tamoxifen treatment, LI and ICR with both post-treatment and changes from baseline of SHBG and all serum biomarkers. We will check whether we need to control for confounding factors and the baseline values. The normality of the models’ residuals will be verified by graphically checking the empirical distribution of the residuals and by looking at Q-Q plots, which compare the cumulative distribution of our data with that of the normal distribution. With multivariate logistic regression, serum biomarkers will be also investigated as categorical variables considering different cut-offs.

To limit the risk of technical bias and take into account the compositional nature of microbiome data, the normalized taxa abundances will be first transformed with the Centred Log-Ratio (CLR) transformation. The most discriminative taxa associated with treatment arms will be selected by using variable selection approaches adapted for compositional data, like Sparse Least Square – Discriminant Analysis (sPLS-DA) and Coda-LASSO. Multivariate linear regression models will be performed to estimate the association between Tamoxifen, LI, ICR and post-treatment clr-abundances of taxa, adjusting for confounders. Associations between post-treatment taxa and consumption of specific groups of foods will be also investigated. The level of physical activity will be quantified with the IPAQ questionnaire. Three categories of physical activity (Low, Moderate, High) will be calculated for each patient following the IPAQ scoring protocol^66^. For QoL assessment, we will analyze the four domains of MenQoL questionnaire: vasomotor, psychosocial, physical and sexual. The overall score of each domain will be calculated by dividing the sum of the domain’s items by the number of items within that domain. Higher domain scores will indicate worse QoL. Associations of the four domains with treatment, LI, ICR and taxa abundances will be estimated considering both post-treatment evaluations (at 3 months and 6 months) and changes from baseline. Multivariate linear or logistic regression will be implemented according to whether the four domains will be analyzed as continuous scales or categorized into classes. The interplay among treatment, serum biomarkers, species (normalized data) associated with treatment arms, diet and QoL will be investigated first with unsupervised methods, like networks based on Spearman’s rank Correlation Coefficient and Canonical Correspondence Analysis (CCA), then with supervised methods for data integration, like block Sparse Least Square-Discriminant Analysis (sPLS-DA) and priority-LASSO hierarchical approaches. To estimate the role of *microbiome* as mediator of treatment arms on SHBG and other biomarkers changes and to investigate its role in mediating the effect of treatments on side effects and QoL, we will perform a mediation analysis based on a counterfactual framework approach. The microbial composition of each patient will be summarized with methods of dimensionality reduction like Principal Component Analysis (PCAPathways data will be preprocessed and analyzed as described for the taxonomic data.

The primary analysis will employ an intention-to-treat approach, which includes all participant irrespective of compliance. An according-to-protocol subgroup analysis will be restricted to women who are “compliant”, such as participants who have taken ≥ 75% of the pills, for thus randomized to tamoxifen and ≥ 75% of the days with the respected caloric restriction, by the reported diary.

## 12.4 Evaluation of Toxicity

All participants will be evaluable for toxicity from the time of randomization.

#

# 13. ETHICAL AND REGULATORY CONSIDERATIONS

##

## 13.1 Institutional Review Board Approval

Prior to initiating the study and receiving agent, the Participating Organizations must obtain written approval to conduct the study from the appropriate IRB. Should changes to the study become necessary, protocol amendments will be submitted to the appropriate IRB for further approval.

## 13.2 Informed Consent

All potential study participants will be given a copy of the IRB-approved Informed Consent to review. The investigator will explain all aspects of the study in lay language and answer all questions regarding the study. If the participant decides to participate in the study, she will be asked to sign and date the Informed Consent document.

Those who refuse to participate or who withdraw from the study will be treated without prejudice.

Participants must be provided the option to allow the use of biological samples, obtained during testing, operative procedures, or other standard medical practices for further research purposes.

Prior to study initiation, the informed consent document must be reviewed and approved by appropriate IRB.

## 13.3 Other

This trial will be conducted in compliance with the protocol, Good Clinical Practice (GCP), and the applicable regulatory requirements.

# 14. FINANCING, EXPENSES, AND/OR INSURANCE

Participants will not be responsible for the costs of this study. Study agent and other study tools will be provided at no cost to the participants. If, as a result of participation in this study, an individual experiences injury from known or unknown risks of the research procedures as described in the informed consent, immediate medical care and treatment, including hospitalization, if necessary, will be available. No monetary compensation is available for the costs of medical treatment for an injury, thus the participant will be responsible for the costs of such medical treatment, either directly or through their medical insurance and/or other forms of medical coverage.

# REFERENCES

**1**. Cuzick J, Sestak I, Bonanni B, et al: Selective oestrogen receptor modulators in prevention of breast cancer: an updated meta-analysis of individual participant data. The Lancet 381:1827–1834, 2013

**2**. Noonan S, Pasa A, Fontana V, et al: A Survey among Breast Cancer Specialists on the Low Uptake of Therapeutic Prevention with Tamoxifen or Raloxifene. Cancer Prevention Research 11:38–43, 2018

**3**. Smith SG, Sestak I, Howell A, et al: Participant-Reported Symptoms and Their Effect on Long-Term Adherence in the International Breast Cancer Intervention Study I (IBIS I). Journal of Clinical Oncology 35:2666–2673, 2017

**4**. Decensi A, Robertson C, Guerrieri-Gonzaga A, et al: Randomized double-blind 2 X 2 trial of low-dose tamoxifen and fenretinide for breast cancer prevention in high-risk premenopausal women. Journal of Clinical Oncology 27, 2009

**5**. Decensi A, Robertson C, Viale G, et al: A Randomized Trial of Low-Dose Tamoxifen on Breast Cancer Proliferation and Blood Estrogenic Biomarkers. JNCI Journal of the National Cancer Institute 95:779–790, 2003

**6**. Guerrieri-Gonzaga A, Sestak I, Lazzeroni M, et al: Benefit of low-dose tamoxifen in a large observational cohort of high risk ER positive breast DCIS. Int J Cancer 139, 2016

**7**. DeCensi A, Puntoni M, Guerrieri-Gonzaga A, et al: Randomized placebo controlled trial of low-dose tamoxifen to prevent local and contralateral recurrence in breast intraepithelial neoplasia. Journal of Clinical Oncology 37, 2019

**8**. Visvanathan K, Fabian CJ, Bantug E, et al: Use of Endocrine Therapy for Breast Cancer Risk Reduction: ASCO Clinical Practice Guideline Update. Journal of Clinical Oncology 37:3152–3165, 2019

**9**. Owens DK, Davidson KW, Krist AH, et al: Medication Use to Reduce Risk of Breast Cancer. JAMA 322:857, 2019

**10**. Hammond GL: Potential functions of plasma steroid-binding proteins. Trends in Endocrinology & Metabolism 6:298–304, 1995

**11**. Fortunati N: Sex Hormone-Binding Globulin: Not only a transport protein. What news is around the corner? J Endocrinol Invest 22:223–234, 1999

**12**. Fortunati N, Catalano M: Sex Hormone-binding Globulin (SHBG) and Estradiol Cross-talk in Breast Cancer Cells. Hormone and Metabolic Research 38:236–240, 2006

**13**. Berrino F, Bellati C, Secreto G, et al: Reducing bioavailable sex hormones through a comprehensive change in diet: the diet and androgens (DIANA) randomized trial. Cancer Epidemiol Biomarkers Prev 10:25–33, 2001

**14**. Harvie MN, Pegington M, Mattson MP, et al: The effects of intermittent or continuous energy restriction on weight loss and metabolic disease risk markers: a randomized trial in young overweight women. Int J Obes 35:714–727, 2011

**15**. Liu N, Feng Y, Luo X, et al: Association Between Dietary Inflammatory Index and Sex Hormone Binding Globulin and Sex Hormone in U.S. Adult Females. Front Public Health 10, 2022

**16**. Hadji P, Kauka A, Bauer T, et al: Effects of exemestane and tamoxifen on hormone levels within the Tamoxifen Exemestane Adjuvant Multicentre (TEAM) Trial: results of a German substudy. Climacteric 15:460–466, 2012

**17**. Johansson H, Puntoni M, Macis D, et al: Abstract PD3-08: Effects of low dose tamoxifen on circulating risk biomarkers in a phase III trial in breast pre-invasive disease. Cancer Res 80:PD3-08-PD3-08, 2020

**18**. Johansson H, Bonanni B, Gandini S, et al: Circulating hormones and breast cancer risk in premenopausal women: A randomized trial of low-dose tamoxifen and fenretinide. Breast Cancer Res Treat 142, 2013

**19**. Masala G, Assedi M, Ambrogetti D, et al: Physical activity and mammographic breast density in a Mediterranean population: The EPIC Florence longitudinal study. Int J Cancer 124:1654–1661, 2009

**20**. Cuzick J, Warwick J, Pinney E, et al: Tamoxifen-Induced Reduction in Mammographic Density and Breast Cancer Risk Reduction: A Nested Case-Control Study. JNCI Journal of the National Cancer Institute 103:744–752, 2011

**21**. Iyengar NM, Arthur R, Manson JE, et al: Association of Body Fat and Risk of Breast Cancer in Postmenopausal Women With Normal Body Mass Index. JAMA Oncol 5:155, 2019

**22**. Serrano D, Pozzi C, Guglietta S, et al: Microbiome as mediator of diet on colorectal cancer risk: The role of Vitamin D, markers of inflammation and adipokines. Nutrients 13, 2021

**23**. Renehan AG, Tyson M, Egger M, et al: Body-mass index and incidence of cancer: a systematic review and meta-analysis of prospective observational studies. The Lancet 371:569–578, 2008

**24**. Iyengar NM, Brown KA, Zhou XK, et al: Metabolic Obesity, Adipose Inflammation and Elevated Breast Aromatase in Women with Normal Body Mass Index. Cancer Prevention Research 10:235–243, 2017

**25**. Longo VD, di Tano M, Mattson MP, et al: Intermittent and periodic fasting, longevity and disease. Nat Aging 1:47–59, 2021

**26**. Harvie MN, Pegington M, Mattson MP, et al: The effects of intermittent or continuous energy restriction on weight loss and metabolic disease risk markers: a randomized trial in young overweight women. Int J Obes 35:714–727, 2011

**27**. Valdemarin F, Caffa I, Persia A, et al: Safety and Feasibility of Fasting-Mimicking Diet and Effects on Nutritional Status and Circulating Metabolic and Inflammatory Factors in Cancer Patients Undergoing Active Treatment. Cancers (Basel) 13:4013, 2021

**28**. Caffa I, Spagnolo V, Vernieri C, et al: Fasting-mimicking diet and hormone therapy induce breast cancer regression. Nature 583:620–624, 2020

**29**. Laws A, Punglia RS: Endocrine Therapy for Primary and Secondary Prevention After Diagnosis of High-Risk Breast Lesions or Preinvasive Breast Cancer. Journal of Clinical Oncology 41:3092–3099, 2023

**30**. DeCensi A, Bonanni B, Maisonneuve P, et al: A phase-III prevention trial of low-dose tamoxifen in postmenopausal hormone replacement therapy users: The HOT study. Annals of Oncology 24, 2013

**31**. He X, Liao Y, Yu S, et al: Sex Hormone Binding Globulin and Risk of Breast Cancer in Postmenopausal Women: A Meta-Analysis of Prospective Studies. Hormone and Metabolic Research 47:485–490, 2015

**32**. Gnagnarella P, Dragà D, Baggi F, et al: Promoting weight loss through diet and exercise in overweight or obese breast cancer survivors (InForma): study protocol for a randomized controlled trial. Trials 17:363, 2016

**33**. Reeves MM, Terranova CO, Eakin EG, et al: Weight loss intervention trials in women with breast cancer: a systematic review. Obesity Reviews 15:749–768, 2014

**34**. Dąbrowska-Galas M, Dąbrowska J, Ptaszkowski K, et al: High Physical Activity Level May Reduce Menopausal Symptoms. Medicina (B Aires) 55:466, 2019

**35**. Barnard ND, Kahleova H, Holtz DN, et al: The Women’s Study for the Alleviation of Vasomotor Symptoms (WAVS): a randomized, controlled trial of a plant-based diet and whole soybeans for postmenopausal women. Menopause 28:1150–1156, 2021

**36**. Vidoni C, Ferraresi A, Esposito A, et al: Calorie Restriction for Cancer Prevention and Therapy: Mechanisms, Expectations, and Efficacy. J Cancer Prev 26:224–236, 2021

**37**. Salvadori G, Mirisola MG, Longo VD: Intermittent and periodic fasting, hormones, and cancer prevention. Cancers (Basel) 13, 2021

**38**. Schübel R, Nattenmüller J, Sookthai D, et al: Effects of intermittent and continuous calorie restriction on body weight and metabolism over 50 wk: A randomized controlled trial. American Journal of Clinical Nutrition 108:933–945, 2018

**39**. Baglietto L, English DR, Hopper JL, et al: Circulating steroid hormone concentrations in postmenopausal women in relation to body size and composition. Breast Cancer Res Treat 115:171–179, 2009

**40**. Varkaneh Kord H, M. Tinsley G, O. Santos H, et al: The influence of fasting and energy-restricted diets on leptin and adiponectin levels in humans: A systematic review and meta-analysis. Clinical Nutrition 40:1811–1821, 2021

**41**. Thomas AM, Manghi P, Asnicar F, et al: Metagenomic analysis of colorectal cancer datasets identifies cross-cohort microbial diagnostic signatures and a link with choline degradation. Nat Med 25, 2019

**42**. Ruo SW, Alkayyali T, Win M, et al: Role of Gut Microbiota Dysbiosis in Breast Cancer and Novel Approaches in Prevention, Diagnosis, and Treatment. Cureus , 2021

**43**. Mantovani A, Allavena P, Sica A, et al: Cancer-related inflammation. Nature 454:436–444, 2008

**44**. Liyanage UK, Moore TT, Joo H-G, et al: Prevalence of Regulatory T Cells Is Increased in Peripheral Blood and Tumor Microenvironment of Patients with Pancreas or Breast Adenocarcinoma 1 [Internet]. 2002Available from: http://journals.aai.org/jimmunol/article-pdf/169/5/2756/1156252/2756.pdf

**45**. Plitas G, Konopacki C, Wu K, et al: Regulatory T Cells Exhibit Distinct Features in Human Breast Cancer. Immunity 45:1122–1134, 2016

**46**. Foulds GA, Vadakekolathu J, Abdel-Fatah TMA, et al: Immune-phenotyping and transcriptomic profiling of peripheral blood mononuclear cells from patients with breast cancer: Identification of a 3 gene signature which predicts relapse of triple negative breast cancer. Front Immunol 9, 2018

**47**. Birzniece V, Sata A, Sutanto S, et al: Neuroendocrine Regulation of Growth Hormone and Androgen Axes by Selective Estrogen Receptor Modulators in Healthy Men. J Clin Endocrinol Metab 95:5443–5448, 2010

**48**. Bonanni B, Serrano D, Gandini S, et al: Randomized biomarker trial of anastrozole or low-dose tamoxifen or their combination in subjects with breast intraepithelial neoplasia. Clinical Cancer Research 15, 2009

**49**. Fabian C, Sternson L, El-serafi M, et al: Clinical pharmacology of tamoxifen in patients with breast cancer: Correlation with clinical data. Cancer 48:876–882, 1981

**50**. Decensi A, Gandini S, Serrano D, et al: Randomized dose-ranging trial of tamoxifen at low doses in hormone replacement therapy users. Journal of Clinical Oncology 25, 2007

**51**. Gnagnarella P, Dragà D, Misotti AM, et al: Validation of a short questionnaire to record adherence to the Mediterranean diet: An Italian experience. Nutrition, Metabolism and Cardiovascular Diseases 28:1140–1147, 2018

**52**. Craig Cl, Marshall Al, Sj,Strm M, Et al: International Physical Activity Questionnaire: 12-Country Reliability and Validity. Med Sci Sports Exerc 35:1381–1395, 2003

**53**. Ainsworth Be, Haskell WL, Herrmann SD, et al: 2011 Compendium of Physical Activities. Med Sci Sports Exerc 43:1575–1581, 2011

**54**. Buffa R, Mereu E, Comandini O, et al: Bioelectrical impedance vector analysis (BIVA) for the assessment of two-compartment body composition. Eur J Clin Nutr 68:1234–1240, 2014

**55**. Piccoli A, Rossi B, Pillon L: Operational equivalence between segmental and whole-body bioelectrical impedance in renal patients. Am J Clin Nutr 59:675–676, 1994

**56**. Holmes CJ, Racette SB: The Utility of Body Composition Assessment in Nutrition and Clinical Practice: An Overview of Current Methodology. Nutrients 13:2493, 2021

**57**. Johansson H, Puntoni M, Macis D, et al: Abstract PD3-08: Effects of low dose tamoxifen on circulating risk biomarkers in a phase III trial in breast pre-invasive disease. Cancer Res 80:PD3-08-PD3-08, 2020

**58**. Yates MS, Coletta AM, Zhang Q, et al: Prospective Randomized Biomarker Study of Metformin and Lifestyle Intervention for Prevention in Obese Women at Increased Risk for Endometrial Cancer. Cancer Prevention Research 11:477–490, 2018

**59**. Bolyen E, Rideout JR, Dillon MR, et al: Reproducible, interactive, scalable and extensible microbiome data science using QIIME 2. Nat Biotechnol 37:852–857, 2019

**60**. Callahan BJ, McMurdie PJ, Rosen MJ, et al: DADA2: High-resolution sample inference from Illumina amplicon data. Nat Methods 13:581–583, 2016

**61**. Janssen S, McDonald D, Gonzalez A, et al: Phylogenetic Placement of Exact Amplicon Sequences Improves Associations with Clinical Information. mSystems 3, 2018

**62**. Quast C, Pruesse E, Yilmaz P, et al: The SILVA ribosomal RNA gene database project: improved data processing and web-based tools. Nucleic Acids Res 41:D590–D596, 2012

**63**. Love MI, Huber W, Anders S: Moderated estimation of fold change and dispersion for RNA-seq data with DESeq2. Genome Biol 15:550, 2014

**64**. Fanti M, Mishra A, Longo VD, et al: Time-Restricted Eating, Intermittent Fasting, and Fasting-Mimicking Diets in Weight Loss. Curr Obes Rep 10:70–80, 2021

**65**. Attinà A, Leggeri C, Paroni R, et al: Fasting: How to Guide. Nutrients 13:1570, 2021

**66**. McDiarmid KP, Wood LG, Upham JW, et al: The Impact of Meal Dietary Inflammatory Index on Exercise-Induced Changes in Airway Inflammation in Adults with Asthma. Nutrients 14:4392, 2022

# APPENDIX A

## LOCAL CO-INVESTIGATORS

**Organization:** **European Institute of Oncology**

Chiara A. Accornero: ChiaraArianna.Accornero@ieo.it

Valentina Aristarco: valentina.aristarco@ieo.it

Gaetano Aurilio: gaetano.aurilio@ieo.it

Enrico Cassano: enrico.cassano@ieo.it

Valeria Dominelli: [valeria.dominelli@ieo.it](mailto:valeria.dominelli@ieo.it)

Irene Feroce: [irene.feroce@ieo.it](mailto:irene.feroce@ieo.it)

Viviana Enrica Galimberti: viviana.galimberti@ieo.it

Patrizia Gnagnarella: patrizia.gnagnarella@ieo.it

Aliana Guerrieri-Gonzaga: aliana.guerrierigonzaga@ieo.it

Costantino Jemos: costantino.jemos@ieo.it

Harriet Johansson: harriet.johansson@ieo.it

Matteo Lazzeroni: matteo.lazzeroni@ieo.it

Debora Macis: debora.macis@ieo.it

Sara Mannucci: sara.mannucci@ieo.it

Emanuela Omodeo Salè: [eomodeo@ieo.it](mailto:eomodeo@ieo.it)

Anna Rotili anna.rotili@ieo.it

Davide Serrano: davide.serrano@ieo.it

Paolo Veronesi: [paolo.veronesi@ieo.it](mailto:paolo.veronesi@ieo.it)

Cristina Zanzottera: cristina.zanzottera@ieo.it

**Organization:** **E.O. Galliera - Ospedale Villa Scassi-Genova ASL3-Regione Liguria**

Mauro D'Amico, [mauro.d.amico@galliera.it](mailto:mauro.d.amico@galliera.it)

Irene Maria Briata, [irene.maria.briata@galliera.it](mailto:irene.maria.briata@galliera.it),

Tania Buttiron Webber, [tania.buttiron@galliera.it](mailto:tania.buttiron@galliera.it)

Stefano Spinaci, [stefano.spinaci@asl3.liguria.it](mailto:stefano.spinaci@asl3.liguria.it)

Maria Giovanna Cossu, [mariagiovanna.cossu@asl3.liguria.it](mailto:mariagiovanna.cossu@asl3.liguria.it)

Nicoletta Gandolfo, [nicoletta.gandolfo@asl3.liguria.it](mailto:nicoletta.gandolfo@as13.liguria.it)

**Organization:** **Istituto Oncologico Veneto (IOV)**

Dott.ssa Maria Teresa Nardi, [mariateresa.nardi@iov.veneto.it](mailto:mariateresa.nardi@iov.veneto.it)

Dott.ssa Sara Watutantrige Fernando, [sara.watutantrigefernando@iov.veneto.it](mailto:sara.watutantrigefernando@iov.veneto.it)

**Organization:** **Istituto Nazionale Tumori G. Pascale**

Buono Giuseppe, [giuseppe.buono@istitutotumori.na.it](mailto:giuseppe.buono@istitutotumori.na.it)

Esposito Emanuela, [emanuela.esposito@istitutotumori.na.it](mailto:emanuela.esposito@istitutotumori.na.it)

Del Mondo Angelo (Stazione Zoologica Anton Dohrn), [angelo.delmondo@szn.it](mailto:angelo.delmondo@szn.it)

Pistelli Luigi (Stazione Zoologica Anton Dohrn), [luigi.pistelli@szn.it](mailto:luigi.pistelli@szn.it)

# Appendix B

## Performance Status Criteria

| **ECOG Performance Status Scale** | | **Karnofsky Performance Scale** | |
| --- | --- | --- | --- |
| **Grade** | **Descriptions** | **Percent** | **Description** |
| **0** | Normal activity. Fully active, able to carry on all pre-disease performance without restriction. | **100** | Normal, no complaints, no evidence of disease. |
|  |  | **90** | Able to carry on normal activity; minor signs or symptoms of disease. |
| **1** | Symptoms, but ambulatory. Restricted in physically strenuous activity, but ambulatory and able to carry out work of a light or sedentary nature (*e.g*., light housework, office work). | **80** | Normal activity with effort; some signs or symptoms of disease. |
|  |  | **70** | Cares for self, unable to carry on normal activity or to do active work. |
| **2** | In bed <50% of the time. Ambulatory and capable of all self-care, but unable to carry out any work activities. Up and about more than 50% of waking hours. | **60** | Requires occasional assistance, but is able to care for most of his/her needs. |
|  |  | **50** | Requires considerable assistance and frequent medical care. |
| **3** | In bed >50% of the time. Capable of only limited self-care, confined to bed or chair more than 50% of waking hours. | **40** | Disabled, requires special care and assistance. |
|  |  | **30** | Severely disabled, hospitalization indicated. Death not imminent. |
| **4** | 100% bedridden. Completely disabled. Cannot carry on any self-care. Totally confined to bed or chair. | **20** | Very sick, hospitalization indicated. Death not imminent. |
|  |  | **10** | Moribund, fatal processes progressing rapidly. |
| **5** | Dead. | **0** | Dead. |

# APPENDIX C

## DIARIO

PID: __ __ __ __ __ __ __ __ __ __ Centro: __ __ __ __ __ Arm N ______

1. Per piacere compili il diario. Le compresse vanno prese a giorni alterni (es. linee grigie o linee bianche), preferibilmente sempre nella stessa fascia oraria (ad es., ora di cena)
2. Se si dimentica un giorno, salti la compressa e passi al prossimo giorno in programma.
3. Segni i giorni in cui decide di fare la restrizione calorica 2 alla settimana.
4. Segni la lettura del contapassi a cadenza settimanale

| Giorno | Data  __/__/____ | Tamoxifen | Restrizione Calorica | Registrazione Contapassi | Note |
| --- | --- | --- | --- | --- | --- |
| 1 |  |  |  |  |  |
| 2 |  |  |  |  |  |
| 3 |  |  |  |  |  |
| 4 |  |  |  |  |  |
| 5 |  |  |  |  |  |
| 6 |  |  |  |  |  |
| 7 |  |  |  |  |  |
| 8 |  |  |  |  |  |
| 9 |  |  |  |  |  |
| 10 |  |  |  |  |  |
| 11 |  |  |  |  |  |
| 12 |  |  |  |  |  |
| 13 |  |  |  |  |  |
| 14 |  |  |  |  |  |
| 15 |  |  |  |  |  |
| 16 |  |  |  |  |  |
| 17 |  |  |  |  |  |
| 18 |  |  |  |  |  |
| 19 |  |  |  |  |  |
| 20 |  |  |  |  |  |
| 21 |  |  |  |  |  |
| 22 |  |  |  |  |  |
| 23 |  |  |  |  |  |
| 24 |  |  |  |  |  |
| 25 |  |  |  |  |  |
| 26 |  |  |  |  |  |
| 27 |  |  |  |  |  |
| 28 |  |  |  |  |  |
| 29 |  |  |  |  |  |
| 30 |  |  |  |  |  |
| 31 |  |  |  |  |  |
| 32 |  |  |  |  |  |
| 33 |  |  |  |  |  |
| 34 |  |  |  |  |  |
| 35 |  |  |  |  |  |
| 36 |  |  |  |  |  |
| 37 |  |  |  |  |  |
| 38 |  |  |  |  |  |
| 39 |  |  |  |  |  |
| 40 |  |  |  |  |  |
| 41 |  |  |  |  |  |
| 42 |  |  |  |  |  |
| 43 |  |  |  |  |  |
| 44 |  |  |  |  |  |
| 45 |  |  |  |  |  |
| 46 |  |  |  |  |  |
| 47 |  |  |  |  |  |
| 48 |  |  |  |  |  |
| 49 |  |  |  |  |  |
| 50 |  |  |  |  |  |
| 51 |  |  |  |  |  |
| 52 |  |  |  |  |  |
| 53 |  |  |  |  |  |
| 54 |  |  |  |  |  |
| 55 |  |  |  |  |  |
| 56 |  |  |  |  |  |
| 57 |  |  |  |  |  |
| 58 |  |  |  |  |  |
| 59 |  |  |  |  |  |
| 60 |  |  |  |  |  |
| 61 |  |  |  |  |  |
| 62 |  |  |  |  |  |
| 63 |  |  |  |  |  |
| 64 |  |  |  |  |  |
| 65 |  |  |  |  |  |
| 66 |  |  |  |  |  |
| 67 |  |  |  |  |  |
| 68 |  |  |  |  |  |
| 69 |  |  |  |  |  |
| 70 |  |  |  |  |  |
| 71 |  |  |  |  |  |
| 72 |  |  |  |  |  |
| 73 |  |  |  |  |  |
| 74 |  |  |  |  |  |
| 75 |  |  |  |  |  |
| 76 |  |  |  |  |  |
| 77 |  |  |  |  |  |
| 78 |  |  |  |  |  |
| 79 |  |  |  |  |  |
| 80 |  |  |  |  |  |
| 81 |  |  |  |  |  |
| 82 |  |  |  |  |  |
| 83 |  |  |  |  |  |
| 84 |  |  |  |  |  |
| 85 |  |  |  |  |  |
| 86 |  |  |  |  |  |
| 87 |  |  |  |  |  |
| 88 |  |  |  |  |  |
| 89 |  |  |  |  |  |
| 90 |  |  |  |  |  |
| 91 |  |  |  |  |  |
| 92 |  |  |  |  |  |
| 93 |  |  |  |  |  |
| 94 |  |  |  |  |  |
| 95 |  |  |  |  |  |
| 96 |  |  |  |  |  |
| 97 |  |  |  |  |  |
| 98 |  |  |  |  |  |
| 99 |  |  |  |  |  |
| 100 |  |  |  |  |  |
| 101 |  |  |  |  |  |
| 102 |  |  |  |  |  |
| 103 |  |  |  |  |  |
| 104 |  |  |  |  |  |
| 105 |  |  |  |  |  |
| 106 |  |  |  |  |  |
| 107 |  |  |  |  |  |
| 108 |  |  |  |  |  |
| 109 |  |  |  |  |  |
| 110 |  |  |  |  |  |
| 111 |  |  |  |  |  |
| 112 |  |  |  |  |  |
| 113 |  |  |  |  |  |
| 114 |  |  |  |  |  |
| 115 |  |  |  |  |  |
| 116 |  |  |  |  |  |
| 117 |  |  |  |  |  |
| 118 |  |  |  |  |  |
| 119 |  |  |  |  |  |
| 120 |  |  |  |  |  |

Firma della partecipante: ________________________________ Data: ________________

Firma del sanitario:______________________________________ Data: ________________

# Appendix D

## Scheda Farmaco

**PID:** __ __ __ __ __ __ __ __ __ __ **Centro: __ __ __ __ __**

**Etichetta:**

|  |  |
| --- | --- |
|  |  |

Firma del medico: __________________ Data: _ _ / _ _ / _ _ _ _

**Numero di Cp date: 120__ Cp rese: □ Si □ No**

**Numero di Cp rese: ________** Data restituzione: _ _ / _ _ / _ _ _

# Appendix E

## Questionari

PID: __ __ __ __ __ __ __ __ __ __ Centro: __ __ __ __ __ Data ____/____/________

## QUESTIONARIO SULL’ATTIVITA’ FISICA

Siamo interessati a conoscere i tipi di attività fisica che le persone fanno come parte della vita quotidiana. Le domande riguarderanno il tempo che lei ha trascorso in attività fisiche negli **ultimi sette giorni**. Cortesemente, risponda ad ogni domanda anche se non si considera essere una persona attiva. Pensi, per favore, alle attività svolte al lavoro, come parte del lavoro svolto in casa ed in giardino, per spostarsi da un luogo all’altro e nel suo tempo libero come divertimento, esercizio fisico o sport.

Pensi a tutte le attività **vigorose**, energiche che ha svolto negli **ultimi sette giorni.** Le attività fisiche **vigorose** sono quelle che richiedono uno sforzo fisico duro e che la fanno respirare con un ritmo molto più frequente rispetto al normale. Pensi *soltanto* a quelle attività fisiche che lei ha svolto per almeno 10 minuti consecutivamente.

1. Durante gli **ultimi sette giorni**, in quanti giorni lei ha svolto attività fisica vigorosa come sollevare oggetti pesanti, zappare, fare aerobica, o pedalare in bicicletta ad una certa velocità?

- **giorni per settimana se n**essuno (**Vada alla domanda 3)**

1. Quanto tempo in totale di solito trascorre in attività fisiche **vigorose** in uno di quei giorni?

- **ore per giorno**  **minuti per giorno** Non sa / non è sicuro/a

Pensi a tutte quelle attività **moderate** che lei ha svolto negli **ultimi sette giorni**. Le attività moderate sono quelle che richiedono uno sforzo fisico moderato e che la fanno respirare con un ritmo un po’ più frequente rispetto al normale. Pensi soltanto a quelle attività fisiche che lei ha svolto per almeno 10 minuti consecutivamente.

1. Durante gli **ultimi sette giorni,** quanti giorni lei ha svolto attività fisica **moderata** come portare pesi leggeri, andare in bicicletta ad un ritmo regolare oppure giocare il doppio a tennis? Non includa il camminare.

- **giorni per settimana** Nessuno **(vada alla domanda 5)**

1. Quanto tempo in totale di solito trascorre in attività fisiche **moderate** in uno di quei giorni?

- **ore per giorno**  **minuti per giorno** Non sa / non è sicuro/a

Pensi al tempo da lei trascorso **camminando negli ultimi sette giorni**. Includa il tempo trascorso sia al lavoro sia a casa, nello spostarsi da un luogo ad un altro e qualsiasi altro cammino che lei ha fatto solo per divertimento, sport, esercizio fisico o per passatempo.

1. Durante gli **ultimi sette giorni,** in quanti giorni lei ha **camminato** per almeno 10 minuti di continuo?

- **giorni per settimana** se nessuno (**Vada alla domanda 7)**

1. Di solito quanto tempo ha trascorso, in uno di quei giorni, **camminando**?

- **ore per giorno**  **minuti per giorno** Non sa / non è sicuro/a

L’ultima domanda riguarda il tempo trascorso stando seduto dal lunedì al venerdì negli ultimi sette giorni. Includa il tempo in cui rimane seduto al lavoro, in casa, nello svolgere un corso di formazione, durante il suo tempo libero. Questo può includere il tempo trascorso alla scrivania, nel far visita ad amici, leggendo, 0 seduto/a sdraiato/a per guardare la televisione.

1. Durante gli ultimi sette giorni, in un giorno della settimana, quanto tempo ha trascorso stando seduto?

**ore per giorno**  **minuti per giorno** Non sa / non è sicuro/a

PID: __ __ __ __ __ __ __ __ __ __ Centro: __ __ __ __ __ Data ____/____/________

## QUESTIONARIO SULLA DIETA

**Buongiorno, Vorremmo farle alcune domande sulla sua dieta abituale.**

Indichi, con una crocetta “X”, il numero di porzioni normalmente consumate per i 15 alimenti o gruppi di alimenti elencati nella tabella sottostante.

Si aiuti con le porzioni di riferimento per identificare la sua frequenza di consumo giornaliera, per gli alimenti elencati dalla domanda n. 1 alla n. 8 e la sua frequenza di consumo settimanale per gli alimenti dalla n. 9 alla n. 16. Se abitualmente consuma una porzione molto piccola o molto grande (rispetto alla porzione di riferimento) dimezzi o raddoppi la frequenza di consumo. Per esempio se normalmente beve mezzo litro di vino al giorno (corrispondenti a circa 4 bicchieri), la frequenza da segnare in tabella sarà “3-4” porzioni al giorno.

È molto importante che risponda a tutte le domande. Nel caso non consumasse qualche alimento, ricordi di fare una crocetta su “mai o raramente”.

**Con quale frequenza ha consumato nell’ultimo mese una porzione dei seguenti alimenti?**

| ALIMENTI | PORZIONE | FREQUENZA DI CONSUMO AL GIORNO | | | | |
| --- | --- | --- | --- | --- | --- | --- |
|  |  | **Mai o**  **raramente** | **Meno di 1 volta /giorno** | **1 volta /giorno** | **2 volte /giorno** | **≥ 3 volte /giorno** |
| 1. Pasta o riso di tipo integrale | **80 gr** | 🞎 | 🞎 | 🞎 | 🞎 | 🞎 |
| 2. Verdura tutti i tipi (sia cruda che cotta) | **200 gr**  **(80 gr insalata)** | 🞎 | 🞎 | 🞎 | 🞎 | 🞎 |
| 3. Frutta tutti i tipi, anche la spremuta fresca | **150 gr** | 🞎 | 🞎 | 🞎 | 🞎 | 🞎 |
| 4. Latte e yogurt | **1 bicchiere/**  **vasetto** (**125 gr)** | 🞎 | 🞎 | 🞎 | 🞎 | 🞎 |
|  |  | **Mai o**  **raramente** | **Meno di 1 volta /giorno** | **1-2 volte /giorno** | **3-4 volte /giorno** | **≥ 5 volte /giorno** |
| 5. Pane e fette di tipo integrale | **1-2 fette**  **(50 gr)** | 🞎 | 🞎 | 🞎 | 🞎 | 🞎 |
| 6. Olio di oliva per cucinare e condire | **1 cucchiaio**  **(10 ml)** | 🞎 | 🞎 | 🞎 | 🞎 | 🞎 |
| 7. Burro, margarina o panna da cucina per cucinare | **1 noce**  **(10 gr)** | 🞎 | 🞎 | 🞎 | 🞎 | 🞎 |
| 8. Vino (bianco e rosso) | **1 bicchiere (125 ml)** | 🞎 | 🞎 | 🞎 | 🞎 | 🞎 |
| ALIMENTI | PORZIONE | FREQUENZA DI CONSUMO ALLA SETTIMANA | | | | |
|  |  | **Mai o**  **raramente** | **Meno di 1 volta /sett** | **1-3 volte /sett** | **4-6 volte /sett** | **≥ 7 volte /sett** |
| 9. Carne rossa (bovino, vitello, maiale), affettati e salumi | **100 gr (carne)**  **50 gr (salumi)** | 🞎 | 🞎 | 🞎 | 🞎 | 🞎 |
| 10. Carne bianca (pollo, tacchino, coniglio) | **100 gr** | 🞎 | 🞎 | 🞎 | 🞎 | 🞎 |
| 11. Formaggi freschi e stagionati | **100 gr (freschi)**  **50 gr (stagionati)** | 🞎 | 🞎 | 🞎 | 🞎 | 🞎 |
| 12. Bevande dolci o gassate (tipo coca-cola, aranciata, gassosa, ecc) | **1 bicchiere**  **(200 ml)** | 🞎 | 🞎 | 🞎 | 🞎 | 🞎 |
| 13. Dolci o pasticcini (non fatti in casa), come torte, biscotti, creme o dolci al cucchiaio | **100 gr** | 🞎 | 🞎 | 🞎 | 🞎 | 🞎 |
|  |  | **Mai o**  **raramente** | **Meno di 1 volta /sett** | **1 volta /sett** | **2-3 volte /sett** | **≥ 4 volte /sett** |
| 14. Pesce (fresco o surgelato) o frutti di mare | **150 gr (pesce)**  **50 gr (frutti di mare)** | 🞎 | 🞎 | 🞎 | 🞎 | 🞎 |
| 15. Frutta secca (noci, mandorle, nocciole) | **1 pugno**  **(30 gr)** | 🞎 | 🞎 | 🞎 | 🞎 | 🞎 |
| 16. Legumi (ceci, lenticchie, piselli, fagioli) | **50 gr (secchi)**  **150 gr (scatola / freschi)** | 🞎 | 🞎 | 🞎 | 🞎 | 🞎 |

**ISTRUZIONI PER LA COMPILAZIONE**

Ogni domanda del questionario si presenta come negli esempi qui sotto:

Nessun Moltissimo

fastidio 0 1 2 3 4 5 6 fastidio

SUDORI NOTTURNI ❒ ❒ - 0 1 2 3 4 5 6

No Sì

Indichi se ha avuto o meno questo problema durante *le ultime 4 settimane*.

SE *NON HA* AVUTO QUESTO PROBLEMA:

Segni "No"

SUDORI NOTTURNI 🗷 ❒ - 0 1 2 3 4 5 6

No Sì

Passi alla domanda successiva.

SE *HA* AVUTO QUESTO PROBLEMA:

Segni "Sì", poi faccia un cerchietto intorno al numero che indica quanto *fastidio* le ha causato questo problema

SUDORI NOTTURNI ❒ 🗷 - 0 1 2 3 4 5 6

No Sì

Passi alla domanda successiva.

Questo questionario è strettamente riservato. Il suo nome non verrà associato in alcun modo alle sue risposte. Comunque, se per qualsiasi motivo non desiderasse rispondere a una domanda, la preghiamo di saltarla e di passare a quella successiva.

**PID:** __ __ __ __ __ __ __ __ __ __ **Centro: __ __ __ __ __ Data ____/____/________**

## QUESTIONARIO SULLA QUALITÀ DI VITA

Indichi se ha avuto ciascuno dei seguenti problemi durante le **ULTIME 4 SETTIMANE**. Se l'ha avuto, valuti quanto ***fastidio*** le ha dato.

Nessun Moltissimo

fastidio 0 1 2 3 4 5 6 fastidio

| 1. | VAMPATE DI CALORE | ❒  No | ❒  Sì | 0 1 2 3 4 5 6 |
| --- | --- | --- | --- | --- |
| 2. | SUDORI NOTTURNI | ❒  No | ❒  Sì | 0 1 2 3 4 5 6 |
| 3. | SUDORAZIONE ECCESSIVA | ❒  No | ❒  Sì | 0 1 2 3 4 5 6 |
| 4. | ESSERE INSODDISFATTA DELLA SUA VITA PERSONALE | ❒  No | ❒  Sì | 0 1 2 3 4 5 6 |
| 5. | SENTIRSI IN ANSIA O AGITATA | ❒  No | ❒  Sì | 0 1 2 3 4 5 6 |
| 6. | AVERE POCA MEMORIA | ❒  No | ❒  Sì | 0 1 2 3 4 5 6 |
| 7. | FARE MENO COSE DI QUANTE ERA ABITUATA A FARNE | ❒  No | ❒  Sì | 0 1 2 3 4 5 6 |
| 8. | SENTIRSI DEPRESSA O GIÙ DI MORALE | ❒  No | ❒  Sì | 0 1 2 3 4 5 6 |
| 9. | AVERE POCA PAZIENZA CON GLI ALTRI | ❒  No | ❒  Sì | 0 1 2 3 4 5 6 |
| 10. | AVERE VOGLIA DI STARE DA SOLA | ❒  No | ❒  Sì | 0 1 2 3 4 5 6 |
| 11. | FLATULENZA (FARE ARIA) O DOLORI DOVUTI ALL’ARIA NELLA PANCIA | ❒  No | ❒  Sì | 0 1 2 3 4 5 6 |
| 12. | DOLORI AI MUSCOLI E ALLE ARTICOLAZIONI | ❒  No | ❒  Sì | 0 1 2 3 4 5 6 |

Nessun Moltissimo

fastidio 0 1 2 3 4 5 6 fastidio

| 13. | SENTIRSI STANCA O SPOSSATA | ❒  No | ❒  Sì | 0 1 2 3 4 5 6 |
| --- | --- | --- | --- | --- |
| 14. | PROBLEMI DI SONNO | ❒  No | ❒  Sì | 0 1 2 3 4 5 6 |
| 15. | DOLORI DIETRO IL COLLO O MAL DI TESTA | ❒  No | ❒  Sì | 0 1 2 3 4 5 6 |
| 16. | CALO DELLA FORZA FISICA | ❒  No | ❒  Sì | 0 1 2 3 4 5 6 |
| 17. | CALO DELLA RESISTENZA FISICA | ❒  No | ❒  Sì | 0 1 2 3 4 5 6 |
| 18. | SENSAZIONE DI MANCANZA DI ENERGIA | ❒  No | ❒  Sì | 0 1 2 3 4 5 6 |
| 19. | AVERE LA PELLE PIÙ SECCA | ❒  No | ❒  Sì | 0 1 2 3 4 5 6 |
| 20. | AUMENTO DI PESO | ❒  No | ❒  Sì | 0 1 2 3 4 5 6 |
| 21. | AUMENTO DELLA PELURIA DEL VISO | ❒  No | ❒  Sì | 0 1 2 3 4 5 6 |
| 22. | CAMBIAMENTI NELL'ASPETTO, NELLA CONSISTENZA O NEL COLORE DELLA PELLE | ❒  No | ❒  Sì | 0 1 2 3 4 5 6 |
| 23. | SENSO DI GONFIORE | ❒  No | ❒  Sì | 0 1 2 3 4 5 6 |
| 24. | DOLORI ALLA PARTE BASSA DELLA SCHIENA | ❒  No | ❒  Sì | 0 1 2 3 4 5 6 |
| 25. | URINARE FREQUENTE-MENTE | ❒  No | ❒  Sì | 0 1 2 3 4 5 6 |
| 26. | PERDITA DI URINA NEL RIDERE O NEL TOSSIRE | ❒  No | ❒  Sì | 0 1 2 3 4 5 6 |

Nessun Moltissimo

fastidio 0 1 2 3 4 5 6 fastidio

| 27. | CAMBIAMENTO DEL DESIDERIO SESSUALE | ❒  No | ❒  Sì | 0 1 2 3 4 5 6 |
| --- | --- | --- | --- | --- |
| 28. | SECCHEZZA VAGINALE DURANTE IL RAPPORTO SESSUALE | ❒  No | ❒  Sì | 0 1 2 3 4 5 6 |
| 29. | EVITARE SITUAZIONI DI INTIMITÀ | ❒  No | ❒  Sì | 0 1 2 3 4 5 6 |

Firma partecipante:_________________________ Data: ___/___/_______
